# Variability in Retinal Neuron Populations and Associated Variations in Mass Transport Systems of the Retina in Health and Aging

**DOI:** 10.3389/fnagi.2022.778404

**Published:** 2022-02-25

**Authors:** Moussa A. Zouache

**Affiliations:** John A. Moran Eye Center, Department of Ophthalmology and Visual Sciences, University of Utah, Salt Lake City, UT, United States

**Keywords:** retina, retinal neurons, mass transport, retinal vasculature, Bruch’s membrane, choriocapillaris, aging, photoreceptors

## Abstract

Aging is associated with a broad range of visual impairments that can have dramatic consequences on the quality of life of those impacted. These changes are driven by a complex series of alterations affecting interactions between multiple cellular and extracellular elements. The resilience of many of these interactions may be key to minimal loss of visual function in aging; yet many of them remain poorly understood. In this review, we focus on the relation between retinal neurons and their respective mass transport systems. These metabolite delivery systems include the retinal vasculature, which lies within the inner portion of the retina, and the choroidal vasculature located externally to the retinal tissue. A framework for investigation is proposed and applied to identify the structures and processes determining retinal mass transport at the cellular and tissue levels. Spatial variability in the structure of the retina and changes observed in aging are then harnessed to explore the relation between variations in neuron populations and those seen among retinal metabolite delivery systems. Existing data demonstrate that the relation between inner retinal neurons and their mass transport systems is different in nature from that observed between the outer retina and choroid. The most prominent structural changes observed across the eye and in aging are seen in Bruch’s membrane, which forms a selective barrier to mass transfers at the interface between the choroidal vasculature and the outer retina.

## Introduction

Increasing age is associated with a broad range of visual impairments that include loss of spatial contrast sensitivity, decreased light and wavelength sensitivities, deficits in the processing of temporal information and slowing of visual processing speed (Zhang et al., 2008; Owsley, 2011). These changes can have dramatic consequences on the quality of life of those affected (Owsley and Burton, 1991; Owsley et al., 1998); however, they remain poorly understood. Improving our understanding of the mechanisms involved in age-related vision impairments is essential to design strategies to slow or even reverse them. It can also help determine characteristics that may be used to differentiate individuals who “age well” – who suffer minimum or manageable vision loss as they age – to those who do not, and, in doing so, assist in identifying precursors of eye diseases (Owsley, 2011).

**FIGURE 1:**
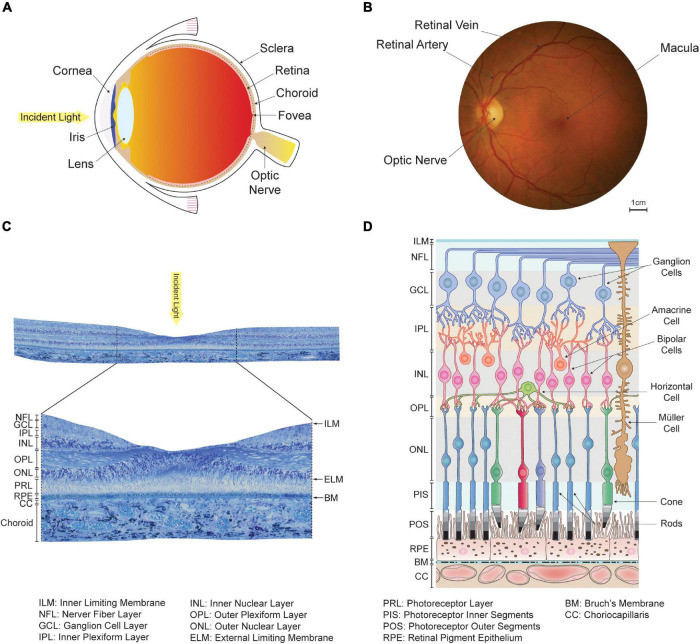
Organization of the human eye and retina. **(A)** Schematic of a human eye. The anterior segments, which include the cornea, iris and lens, direct light toward the retina located in the back of the eye. **(B)** En-face view of the retina captured in a healthy individual using an ophthalmoscope. The optic nerve and large retinal arteries and veins are visible, but the underlying choroidal vasculature is not. The fovea, a region of the retina specialized for high-acuity and color vision, lies at the center of the approximately 5.5 mm wide macula. **(C)** View of a transverse section of the retina taken from a human donor eye in the macula. Histologically, the retina appears as layered tissue formed by retinal neurons, endothelium, and glial cells. **(D)** Schematic of the cellular organization of the retina and choriocapillaris, adapted and modified with permission from Campello et al. (2021). The location of histologically defined retinal layers is indicated. The outer retina consists of the retinal pigment epithelium and photoreceptor outer and inner segments. The inner retina includes horizontal, bipolar, amacrine and ganglion cells, which are all involved in the processing of signals originating from the photoreceptors.

Every part of the eye undergoes changes as we age (Owsley, 2011; Grossniklaus et al., 2013). The human eye, like that of other vertebrates, essentially consists of concentric layers of tissue enclosing a fluid-filled chamber (see Figure 1A). The primary function of the cornea, iris, and lens located in the anterior part of the eye is to focus and direct light toward the posterior segments, where the retina lies (Zouache et al., 2016b); see Figure 1B. It is there that photoreceptor cells perform phototransduction, or the conversion of photons into electrochemical impulses. Signals from the photoreceptors are received and processed by an intricate machinery of neurons and turned into action potentials carried by the axons of approximately one million retinal ganglion cells (see Figures 1C,D). These axons run along the inner surface of the retina before converging into the optic nerve, which travels to the brain (Oyster, 1999; Masland, 2012).

While many age-related vision impairments are driven by transmission losses in the optical media of the eye (Hockwin and Ohrloff, 1984; Reim, 1984; Rohen and Lütjen-Drecoll, 1984; Owsley and Burton, 1991; Pierscionek, 1996), cellular and molecular changes occurring within the retina also play an important role (Weale, 1986; Marshall, 1987; Grossniklaus et al., 2013; Campello et al., 2021). Retinal senescence is likely driven by a complex series of changes that affect multiple cellular and extracellular elements that interact with each other (Marshall, 1987). However, our understanding of normal interactions between retinal elements and processes necessary for tissue function and survival is considerably limited. The role that these interactions and their alterations play in retinal aging is therefore not fully appreciated, which considerably restricts our ability to identify strategies to effectively slow or prevent visual changes associated with increasing age.

**FIGURE 2:**
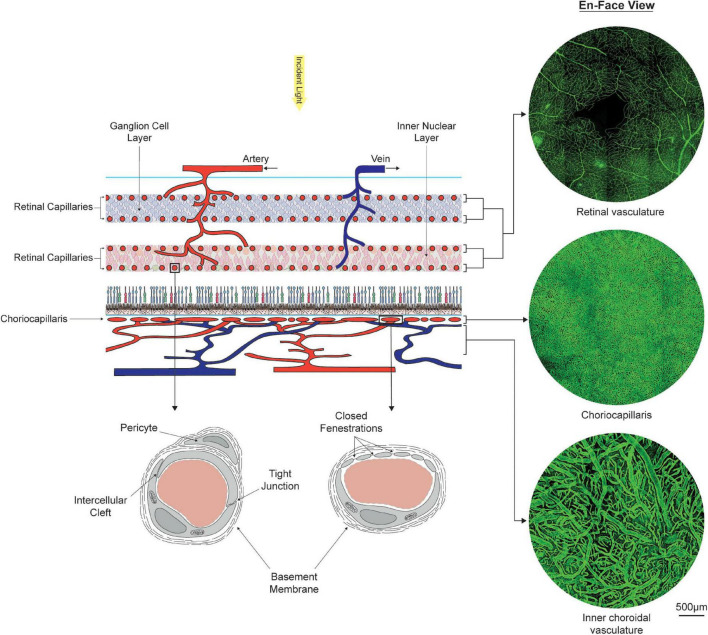
Organization, morphology, and ultrastructure of the dual circulatory systems of the retina formed by the retinal and choroidal vasculatures. The inner two-thirds of the retina is sustained by at least four layers of retinal capillaries, which connect alternating arterial and venous branches. Oxygen and nutrients are supplied to the outer one third of the retina by the choriocapillaris, located externally to the retinal pigment epithelium. En-face visualizations of the retinal and choroidal vasculatures at the fovea were obtained by immunostaining portions of retinas and choroid with Ulex Europaeus Agglutinin and imaging them using confocal microscopy. Stark differences in vascular density between the choriocapillaris and the retinal capillary bed can be observed. Ultrastructural differences are also present. Retinal capillaries form a tightly regulated barrier between blood and tissue. Capillaries from the choriocapillaris present with closed fenestrations, which facilitate the transfer of small and large molecules.

The purpose of this review is to assess how documented spatial and age-related variations in retinal neuron populations relate to changes observed in the structure and function of the mass transport systems to the retina. Homeostasis, metabolism, and survival of retinal cells rely on the adequate supply of metabolism substrates and clearance of metabolic waste products from the retina. These processes are supported by a dual circulatory system formed by the choroidal and retinal vasculatures (see Figure 2). The choroidal vasculature and its microvascular bed, the choriocapillaris, support the metabolic requirement of the outer half of the retina, which is mainly composed of photoreceptors and a monolayer of epithelial cells. The inner part of the retina, which mainly consists of neuronal and glial cells, is sustained by the retinal vasculature (Wong-Riley, 2010).

An underlying hypothesis to this work is that age-related changes in the structure and function of the mass transport systems sustaining the retina correlate with variations in retinal cell populations observed in aging. This would support the idea that the transport of material that is key to healthy cell metabolism is adjusted in aging, so that correlated changes in the retina and its metabolite delivery system are adaptive. Departure from this correlated behavior may then put the eye at a higher risk of vision impairments and disease. The first step in testing this hypothesis is to determine the nature of the relation between retinal neuron populations and mass transport systems in health. The human eye offers an ideal template to explore this relationship. Spatial variations in the structure of the retina and choroid are well-documented and, within an adequate framework, may be harnessed to identify correlated patterns of change. Aging can provide valuable insights into the resilience of correlated behaviors to perturbations occurring over large timescales. Changes in the relation between retinal neurons and their respective metabolite delivery system in aging are therefore also explored.

## Framework for Investigation

This review investigates biological systems – retinal neurons and their respective metabolite delivery systems – often considered in isolation. An important factor to consider when assessing relations between any biological systems is the characteristic length- and timescales associated with the elements, systems, and processes at play (Lesne, 2013).

### Scale and Interactions

Many interactions between retinal cells and the retinal and choroidal vasculatures occur through a movement of molecules. For instance, the movement of oxygen, nutrients, metabolism byproducts, signaling proteins and growth factors between vasculatures and neurons partly determines the metabolism of these cells, their function, and their ability to survive and adapt to changes in their microenvironment. Exchange between neurons and the retinal and choroidal vasculatures may be studied at the scale of cells, tissue or even organs (see Figure 1). Phenomena associated with each scale provide different – and sometime overlapping – information on the state of retinal components. For instance, quantum mechanics may be better suited to describe phototransduction (Sia et al., 2014) whereas stochastic kinetics is more appropriate to model chemical kinetics and generate reaction constants (Lecca, 2013). As separate models may be applied to understand and describe processes occurring at different scales, the challenge becomes to integrate them into a framework capable of capturing the interplay between them (Lesne, 2013, 2007; Green and Batterman, 2017).

Advances in molecular techniques have made it possible to explore variations in genome, epigenome, transcriptome, metabolism and immune response in the retina of human donor eyes (Campello et al., 2021). These methods have the potential to provide a resolution sufficient to dissect spatial and temporal changes such as the ones observed in aging up to the level of a cell (Wang S. et al., 2020). Beyond the theoretical and technical issues associated with the processing and analyses of these large datasets (Mattmann, 2013; Leonelli, 2019; Teschendorff, 2019), the characteristic length-scale associated with these methods is too small to extract data pertaining to interactions between cells and extracellular components. For instance, transcription in photoreceptors is partly determined by external stimuli, some of which result from chains of events involving the choroidal vasculature. However, the choroid is far upstream (or downstream) in this chain of event; measuring its effect on photoreceptors may not be possible because of processes involving other retinal components. It is therefore difficult to detect the effect of the choroidal vasculature on photoreceptors at this scale. At the other end of the spectrum of length-scales, interactions between retinal neurons and the retinal vasculature are impossible to characterize at the organ level as this scale is too large to consider them as distinct entities.

To ensure that most of the information relevant to interactions between retinal neurons and their respective metabolite delivery systems is captured, this review focuses on structures and processes at the cellular and tissue scales.

### From Geometry to Mass Transport

Capturing variations in the structures and processes determining mass exchange at the cellular and tissue levels experimentally is challenging. Our understanding comes mostly from cross-sectional analyses of human donor eyes (Oyster, 1999), which only provide snapshots of variations across the eye and over time. In addition, these analyses are fundamentally limited by the scarcity of human donor eyes in certain age groups. Animal and *in vitro* models have been used to attempt to address this limitation (Conn, 2006); however, the human retina has singular properties that render extrapolation from these systems difficult (Hussain et al., 2010; Kam et al., 2019). In this review, we harness the fact that local mass transport is partly determined by the morphology of cells and the specific geometry of their interface with other tissue components. We can therefore ascertain that variabilities in the structure of the retina provide an indirect way of assessing variations in transport processes. This approach is in some ways imperfect, as structures alone are often insufficient to describe mass transport within any biological system. A good understanding of the fundamental laws governing transport phenomena and how specific structures influence them is necessary to draw any interpretation (Lighthill, 1972). This understanding often comes from experimental and theoretical models developed specifically to study well-defined systems and requires careful considerations centered around basic principles of mass transfers.

### Fundamentals of Mass Transfers for Cells and Tissues

Within blood vessels, molecules are transported through a combination of advection and diffusion. Diffusion is the net movement of material along gradients of concentration. Advection is the movement of material due to the motion of a fluid; it is the dominant transport mechanism in blood vessels. Advection is a more efficient mode of transport, and its prominence with respect to diffusion is determined by the balance between pressure gradients along arteries, capillaries and veins that drive blood flow and factors effectively creating a resistance to this flow. Most of this vascular resistance is caused by the diameter of blood vessels and the viscosity of blood (Lighthill, 1972). The Hagen-Poiseuille law, which applies to incompressible uniform viscous fluids flowing through cylindrical tubes (Batchelor, 2000), is commonly used to describe the salient features of the flow associated with the specific geometry of blood vessels (Lighthill, 1972; Pournaras et al., 2008). This approximate law states that blood velocity varies as *R*^2^ and that the blood flow rate varies as *R*^2^Δ*P*/μ*L*, where *R* and *L* are the radius and length of the vessel considered, respectively, Δ*P* is the pressure difference between the entry and exit of the vessel and μ is the viscosity of blood. Blood velocity is minimal within capillaries, where vessel radii are smallest and most of the exchange between plasma and tissue occur.

**FIGURE 3:**
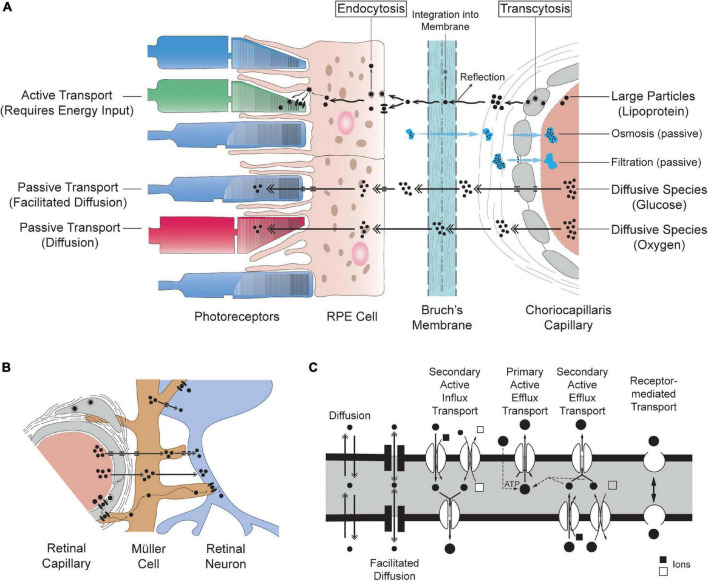
Schematic representation of mass transport systems of the retina. The movement of molecules shown in **(A,B)** is directed from vasculatures to tissue only for simplicity; although, displayed mechanisms are also applicable to the opposite direction. **(A)** Simplified schematic of the transfer of material across choriocapillaris endothelial cells, Bruch’s membrane, RPE cells and photoreceptors (not to scale). **(B)** Mass transfers in the inner retina illustrating the symbiotic relationship between retinal endothelial cells, glial cells (here a Müller cell) and retinal neurons. **(C)** Schematic of the mechanisms of transport across cells adapted and modified from Hosoya and Tachikawa (2012). Transport is traditionally classified as passive (diffusion), carrier-mediated (facilitated diffusion, primary active efflux and secondary active influx and efflux) or receptor-mediated. The conventions used to depict mechanisms of transport in **(A,B)** are consistent with **(C)**.

Molecules contained in plasma may cross blood vessel walls through passive or active transport. Passive transport does not require any input of energy from the cell and instead relies on the tendency of molecules to travel from regions of low concentration to regions of high concentration. The main types of passive transport across cells are diffusion, facilitated diffusion (diffusion mediated by transport proteins embedded in plasma membranes), filtration and osmosis (see Figure 3). The transfer of oxygen from blood to tissue relies solely on molecular diffusion, whereas glucose reaches tissue through facilitated diffusion (by way of transporters including GLUTs) (Mantych et al., 1993). Filtration consists of the movement of water and soluble molecules along gradients of hydrostatic pressure. Active transport typically involves a movement of molecules from regions of high concentration to regions of low concentration (thus ensuring that concentration gradients are maintained). It requires energy input from the cell in the form of adenosine triphosphate (ATP) or membrane potential and may also involve moving material through transendothelial channels or vesicles (also called caveolae). Macromolecules including lipids, insulin and albumin typically cross endothelial cells through active transport (Simionescu et al., 2002). Passive transport across microvascular beds has been extensively studied both experimentally and theoretically. Active transport is tightly linked to several interdependent factors including cellular metabolism, microenvironment and external stimuli and is therefore often more complex to model as compared to passive transport. Our understanding of active transport across capillaries relies primarily on classic experimental studies of microvascular permeability to lipid-insoluble endogenous and non-endogenous macromolecules (Simionescu et al., 2002; Sarin, 2010).

The movement of molecules across capillaries is partly determined by the physical characteristics of structures composing them such as the thickness of their basement membranes and the diameter and spatial distribution of inter-endothelial junctions. The molecular composition of the extravascular space, the structure of cells consuming or delivering transported material and their distance from vascular compartments also influence molecular transfers significantly. At a basic tissue scale (∼1 mm), the movement of material is generally described using average statistics based on mean squared displacements of molecules. Within this framework, the effect of structures influencing the movement of material – such as the geometry of capillaries and composition of the extravascular space – is described using averaged characteristics, such as diffusivity, permeability and cellular density. These measures translate the underlying small-scale structure of molecules, cells and media across which transfers occur; they may vary in space and time and are often determined experimentally. Within frameworks using averaged statistics (in tissue level models for instance), the transfer of diffusive molecules between the vasculature and tissue is mainly a function of blood flow, the geometry of capillaries, the concentration of a specific compound in blood and its consumption in tissue (which is generally a function of the number of cells consuming this compound per volume of tissue) and the resistance of the capillary wall, extravascular space and cells to diffusion (Keener and Sneyd, 2009; Zouache et al., 2019). The topology of a vascular bed is an important factor to consider when assessing its capacity for tissue perfusion (LaBarbera, 1990; West et al., 1997; Zouache et al., 2016a). Another element often examined is the vascularity – or vascular density – of a vascular bed. This parameter describes the fraction of tissue occupied by blood vessels and is calculated over a closed volume. Variations in vascular density give insights into spatial differences in metabolite supply and tissue energy requirements.

### Barriers to Transfers

In the retina, the transfer of metabolism substrates and by-products to and from blood must be adequately controlled and regulated. This is the main function of the blood-retinal barrier (BRB), which involves several cellular and extracellular structures regulating transfers between retinal capillaries and the inner part of the retina (inner BRB, iBRB) and between the choriocapillaris and the outer part of the retina (outer BRB, oBRB) (Cunha-Vaz, 1976). Movement across these barriers is generally described within frameworks that place the cell at the center of all transport processes. While the terminology employed may differ, this cell-centric description is entirely encompassed in the fundamental framework described in Section “Fundamentals of Mass Transfers for Cells and Tissues.” Within the cell-centric framework, the exchange of molecules is described in terms of transcellular and paracellular pathways (Pournaras et al., 2008; Nakanishi et al., 2016). The movement of water, small nutrients, ions, and macromolecules across endothelial cells occurs through diffusion, carrier mediated or receptor-mediated transport (see Figure 3C), which involve both passive and active types of transport. Most proteins are non-selectively transported across endothelial cells in vesicles (carrier-mediated transport) (Feng et al., 1996; Simionescu et al., 2002; Minshall et al., 2003; Stan, 2005; Mehta and Malik, 2006). The paracellular pathway allows for passive transport across the space separating endothelial cells and is modulated by intercellular adhesion and the structure of intercellular clefts (see Figure 2). Tight junctions form size- and charge-selective semipermeable barriers to diffusion (Van Itallie and Anderson, 2004; Campbell and Humphries, 2012; Zihni et al., 2016).

The extracellular structures involved in the regulation of transfers between blood and tissue are the glycocalyx and extracellular matrix. The glycocalyx consists of a coat of macromolecules bound to the apical and luminal plasma membrane of epithelia and endothelial cells (Möckl, 2020). Through its molecular structure it acts as a charge-selective barrier to plasma membranes. It affects oncotic pressure gradients driving capillary filtration (Mehta and Malik, 2006) and may regulate protein diffusion (Freeman et al., 2018). Another important function of the glycocalyx is to attenuate the effect of mechanical forces caused by the flow of blood on endothelial cells and thus to preserve their function (Gouverneur et al., 2006). The extracellular matrix consists of the assembly of many components secreted by surrounding cells. Its composition regulates transport in and out of cells, and therefore influences cellular homeostasis and cell-to-cell signaling (Hubmacher and Apte, 2013).

### Experimental Data

Many experimental methods have been applied to characterize retinal and choroidal structures and processes relevant to the study of retinal mass transport systems at the cellular and tissue levels. Investigations using histology rely on a variety of methods to process and image tissue dissected from human eyes. These studies may be limited by tissue availability and the difficulty of phenotyping donor eyes for diseases of the anterior or posterior segments. Variability in times to fixation and processing methods may cause morphological changes that need to be accounted for Tran et al. (2017). Differences in the location and size of the samples analyzed can significantly limit comparisons between studies. Inconsistencies between studies using histology may also be due to the lack of correction for multiple counting of cells on sections and for tissue shrinkage (Curcio et al., 1990; Harman et al., 1997). The absence of consensus on diagnostics and post-processing methods (Garza-Gisholt et al., 2014) can also explain discrepancies between findings. *In vivo* imaging techniques include optical coherence tomography (OCT), optical coherence tomography angiography (OCTA) and adaptive optics (AO). OCT uses low-coherence interferometry to generate cross-sectional images (B-scans) of optical scattering from retinal structures with a longitudinal and lateral spatial resolution of a few micrometers (Huang et al., 1991). By serially recording B-scans, OCTA captures variations in the intensity and phase of backscattered light due to intrinsic movement within tissue, which comes mainly from erythrocytes in blood vessels (Kashani et al., 2017). OCTA has been used to visualize and quantify the retinal (Coscas et al., 2016; Iafe et al., 2016; Garrity et al., 2017; Lavia et al., 2019) and choroidal (Maruko et al., 2018; Wang et al., 2018; Zheng et al., 2019; Wang E. et al., 2020) vasculatures, including in aging (Spaide, 2016; Wei et al., 2017; Lavia et al., 2019; Zheng et al., 2019) and disease (Laíns et al., 2021). OCT and OCTA provide valuable tissue-level information on the retina; however, the lateral resolution that they offer is not sufficient to image individual cells. AO systems have so far mostly been used in research settings. A large number of groups robustly image and quantify the retina at the cellular and sub-cellular levels using AO, with instrumentation capabilities varying according to applications (Marcos et al., 2017). AO-based systems have been applied to image a variety of neurons including cones (Chui et al., 2008a,b; Song et al., 2011; Zhang et al., 2015), rods (Dubra et al., 2011; Merino et al., 2011; Wells-Gray et al., 2018), and ganglion cells (Liu et al., 2017); subretinal structures such as retinal pigment epithelial cells (Liu et al., 2016) and choriocapillaris (Kurokawa et al., 2017); normal and remodeled retinal vasculature (Chui et al., 2016; Sapoznik et al., 2018); and structures and processes associated with various pathologies of the retina (Langlo et al., 2014; Querques et al., 2014; King et al., 2017; Zhang et al., 2017; Karst et al., 2019; Miller et al., 2019; Hammer et al., 2020). Blood flow in the retinal and choroidal vasculatures has been assessed using several dye dilution techniques such as fluorescent dye angiography and scanning laser ophthalmoscope angiography. Dye dilution techniques rely on the injection of a dye into the general circulation and its visualization as it travels through and across blood vessels (Wei et al., 2018). Extractable information includes structural characteristics of blood vessels and average travel time, which has a dependence on the diffusivity of the dye used (Zouache et al., 2016a).

## Cellular Organization of the Retina

The retina is composed of three classes of cells – neurons, glial cells and epithelial cells – organized in four distinct layers; see Figure 1C. Its outermost layer is the retinal pigment epithelium (RPE), which consists of a continuous monolayer of approximately hexagonal pigmented cells. The neural retina lies internally to the RPE and is formed by three nuclear layers enclosing neuronal cell bodies and two plexiform layers of synapses (see Figure 1D; Oyster, 1999).

### Neuronal Organization

General classifications of retinal neurons are based on morphological and physiological analyses of human tissue. They are consistent with methods based on single-cell RNA sequencing (Liang et al., 2019; Menon et al., 2019; Peng et al., 2019; Orozco et al., 2020; Yan et al., 2020; Yi et al., 2020). The outermost layer of the neural retina is formed by approximately 100 million photoreceptors arranged in a continuous array. Approximately 95% of these cells are rods (Curcio et al., 1990), which use rhodopsin as a pigment and are specialized for vision in dim light. The remaining photoreceptors consist of three types of cones functionally defined by the opsin that they express (Masland, 2012; Hoon et al., 2014). Cones are either sensitive to short-, medium- or long-wavelengths. They are approximately 100 times less sensitive to light than rods and are better adapted for bright-light and high acuity color vision (Hoon et al., 2014). Light is transduced in the outer segments, where photopigments are located; photoreceptor inner segments contain mitochondria, and the outer nuclear layer is made up of photoreceptor nuclei (Oyster, 1999). Photoreceptors synapse onto bipolar and horizontal cells at the outer plexiform layer. The role of these neurons is to modify and edit photoreceptor signals before communicating them to ganglion cells. The retina contains at least twelve types of bipolar cells, each with a unique physiology. Multiple types of bipolar cells are connected to cones; however, only one type is connected to rods (Strettoi et al., 2010; Masland, 2012). Horizontal cells modulate synaptic transmissions between rods and cones and bipolar cells. Widespread synaptic connections emphasize differences in signals between photoreceptor cells by providing inhibitory feedback to rods and cones and possibly bipolar dendrites (Masland, 2012). The streams of information carried by bipolar cells are reorganized at the inner plexiform layer and sampled by ganglion cells under refinement from amacrine cells. The body of ganglion cells along with some displaced amacrine cells form the ganglion cell layer. Ganglion cells integrate the processed signals from bipolar and amacrine cells and convey information to the brain. Approximately 1% (La Morgia et al., 2010) of ganglion cells express the protein melanopsin, which makes them intrinsically photosensitive. With a sensitivity and spatial resolution inferior to those of rods and cones, these cells are mainly involved in non-image-forming vision. They play a key role in contrast detection and modulate many responses to light such as circadian entrainment and the pupillary light reflex (Mure, 2021). The innermost layers of the retina consist of the nerve fiber layer, which contains the axons of the ganglion cells, and the inner limiting membrane, which is composed of terminal expansions of Müller cells extending from the photoreceptor layer (Oyster, 1999).

### Glial Cells

The retina contains several types of glial cells that provide structural support to retinal neurons and help maintain retinal homeostasis and retinal integrity (Goldman, 2014; Vecino et al., 2016; Jäkel and Dimou, 2017). Ninety percent of these glial elements are Müller cells. These radially oriented cells extend from the inner limiting membrane to the external limiting membrane, where they form junctions with photoreceptor inner segments (Goldman, 2014; Vecino et al., 2016). Müller cells are not involved in the processing of vision. Their size and number [they are estimated to account for up to 15% of the volume of the retina (Oyster, 1999)], their dense and regular pattern and their close proximity with retinal neurons indicate that they constitute an anatomical and functional intermediary between neurons and compartments with which they exchange molecules (Reichenbach and Robinson, 1995; Bringmann et al., 2006; Vecino et al., 2016). Astrocytes are another type of glial cells present in the human retina. Almost exclusively confined to the innermost retinal layers, their presence and distribution is correlated with that of blood vessels. Astrocyte processes extend to both blood vessels and neurons. Their main functions are to provide enhanced support for degenerating axons and to help maintain the blood-retinal barrier (Vecino et al., 2016).

### Retinal Pigment Epithelium

While not involved in the neuronal processing of vision (Strauss, 2005), very few cells of the eye perform as many different functions as the RPE. This monolayer of approximately hexagonal cells separates the retina from the endothelium of the underlying choriocapillaris. The RPE plays a critical role in the normal functioning of photoreceptors by eliminating water from the subretinal space, performing the phagocytosis of photoreceptor outer segment membranes and supplying essential nutrients to the photoreceptors through epithelial transport. In doing so, it impacts on the kinetics of the chemical reactions occurring during the visual cycle significantly (Marmor and Wolfensberger, 1998; Strauss, 2005).

### Blood Supply

The retinal vasculature sustains the region of the retina extending approximately from the outer nuclear layer to the inner limiting membrane. The remaining outer retina, which includes the photoreceptor inner and outer segments and the RPE, is supported by the choroidal vasculature (see Figure 2). These two vascular systems arise from the ophthalmic artery, which branches into the central retinal artery supplying the retinal vasculature and into the medial and lateral posterior ciliary arteries supplying the choroid (Hayreh, 1962, 1963; Hayreh and Dass, 1962). Neither the retinal nor the choroidal vasculature can compensate for the loss of the other, so that the retina relies on both for survival (Oyster, 1999).

### Metabolism of the Retina

The visual system is the highest energy-consuming system of the brain (Niven and Laughlin, 2008). Impaired energy metabolism causes visual deficits (Linsenmeier and Zhang, 2017) and may be to blame in the pathogenesis of degenerative vitreoretinal disorders (Léveillard et al., 2019). Energy necessary for cellular function is transferred in the form of adenosine triphosphate (ATP). This high-energy molecule is produced through glycolysis or oxidative phosphorylation. Glycolysis takes place in the cytosol. It converts glucose into pyruvate, generating two molecules of ATP in the process. Oxidative phosphorylation occurs in the mitochondria and uses pyruvate as a substrate. It requires the presence of oxygen and produces up to 36 molecules of ATP for each molecule of glucose consumed. When oxygen is limited, oxidative phosphorylation is hindered and the pyruvate produced through glycolysis is reduced to lactate (Alberts et al., 2014). As in the brain, retinal neurons use glucose as their main energy substrate and are dependent on the more energetically effective oxidative phosphorylation to generate ATP. They are therefore very sensitive to fluctuations in glucose and oxygen supplies (Erecińska and Silver, 2001; Linsenmeier and Zhang, 2017). ATP supports most neuronal functions, including protein and neurotransmitter syntheses and recycling. Active transport of ions against their electrical and concentration gradients is the largest energy-consuming neuronal functions (Wong-Riley, 2010). In contrast with retinal neurons, glial cells rely mainly on glycolysis for their ATP needs (Winkler et al., 2000).

Photoreceptors have one of the highest metabolic rates of any cell of the human body (Linsenmeier and Braun, 1992; Niven and Laughlin, 2008; Wong-Riley, 2010). Most of the ATP used by photoreceptors is produced through oxidative phosphorylation, which occurs mainly in the mitochondria-rich inner segments. It is there that most of the oxygen diffusing from the choriocapillaris and retinal circulation is consumed. Studies performed on monkeys indicate that oxygen consumption is larger in the perifovea as compared to the fovea (Yu et al., 2005; Birol et al., 2007). Oxygen consumption is also significantly greater in dark-adapted condition than under illumination (Birol et al., 2007). While many aspects of their normal metabolism remain to be fully understood, it is now well established that photoreceptors generate large amounts of lactate in the presence of oxygen through glycolysis (aerobic glycolysis) (Lindsay et al., 2014; Du et al., 2016a; Chinchore et al., 2017). In fact, it has been estimated that between 80 and 90% of glucose molecules used by adult photoreceptors is consumed through aerobic glycolysis alone. This pathway generates intermediates necessary to the formation of large molecules involved in the visual process (Narayan et al., 2017). The large amounts of lactate produced by photoreceptors may also fuel both Müller and RPE cells. Lactate has been shown to suppress glucose consumption in the RPE, with the effect of increasing the amount of the molecule reaching photoreceptors (Kanow et al., 2017). RPE cells are specialized for reductive carboxylation, a type of metabolism that heavily relies on mitochondria (Du et al., 2016b).

## Delivery System to Inner Retinal Neurons

The inner retinal mass transport system supports the metabolism of many retinal neurons including ganglion, bipolar, horizontal and amacrine cells. Highly regulated, it relies on an adequate blood supply to retinal capillaries in different regions of the retina.

### Retinal Vasculature

Upon branching from the ophthalmic artery, the central retinal artery travels within the optic nerve and inserts into the retina through the optic nerve head. There, it divides into large superior and inferior branches, which further ramify into dependent branches radiating across the retinal surface (Hayreh, 1962, 1963; Hayreh and Dass, 1962). The basic network topology of the retinal vasculature consists of a tree, where blood may only follow a limited number of anatomical routes determined by the branching pattern of arteries of veins (nodes of the tree). Terminal arteries (arterioles) branch from parent vessels at an approximately right angle (Pournaras et al., 2008). *In vivo* measurements indicate that the relation between blood flow rate and diameter among retinal arteries and veins is consistent with Poiseuille’s flows (Riva et al., 1985; Feke et al., 1989); although the velocity profile is flatter rather than parabolic in larger arteries and veins (Zhong et al., 2011). Throughout the retina, larger vessels remain close to the inner limiting membrane. Across most of the retina arteries and veins alternate, so that one vein typically lies between two consecutive arteries (Stokoe and Turner, 1966; Wise et al., 1975); although they tend to be dissociated in the periphery of the retina (Stokoe and Turner, 1966). Capillary beds connect consecutive arterial and venular branches, forming an interconnecting network arranged in a multi-layer pattern, each of them supplying distinct sets of neurons (see Figure 2). Retinal veins merge into the central retinal vein, which exit the eye through the optic nerve head and joins the superior ophthalmic vein (Hayreh, 1962, 1963; Hayreh and Dass, 1962).

### Retinal Vascular Pattern

Analyses based on histology (Snodderly et al., 1992; Pournaras et al., 2008; Chan et al., 2012; Tan et al., 2012), OCT (Chan et al., 2015), OCTA (Campbell et al., 2017; Muraoka et al., 2018; Hormel et al., 2020) and AO (Kurokawa et al., 2012) indicate that the retinal capillary network is arranged in up to four layers (or plexuses) depending on location. Supplied by the central retinal artery, the superficial vascular plexus consists of a network of arteries, arterioles, capillaries, venules and veins contained primarily within the ganglion cell layer. Intermediate and deep capillary network line the inner and outer sides of the inner nuclear layer, respectively, and support the metabolic requirements of amacrine cells, bipolar cells and horizontal cells. These plexuses are supplied by anastomoses from the superficial vascular network (Snodderly et al., 1992; Provis, 2001) and have a lobular organization. The dense radial peripapillary plexus seen in the proximity of the optic nerve head and part of the macula and posterior pole forms a fourth vascular plexus that sustains the densely packed nerve fiber layer bundles (Michaelson, 1956; Kornzweig et al., 1964; Henkind, 1967; Jia et al., 2014; Campbell et al., 2017). Capillaries composing it are supplied and drained by a small number of arterioles and venules from the superficial vascular plexus and run parallel to axons from the nerve fiber layer (Henkind, 1967; Alterman and Henkind, 1968; Campbell et al., 2017; Muraoka et al., 2018). Close to the fovea, capillary plexuses merge into a single layer of capillaries that delineate a region of the retina deprived of blood vessels, the foveal avascular zone (FAZ) (Campbell et al., 2017; Nesper and Fawzi, 2018).

The nature of the connections between capillary plexuses is a key determinant of blood flow patterns in the retinal vasculature and mass exchange with retinal neurons. Animal experiments indicate that venous drainage may predominantly occur through the deep vascular network (Fouquet et al., 2017). While some controversy remains, evidence suggests that the organization of retinal capillaries is neither serial nor parallel (Hormel et al., 2020). The various retinal vascular plexuses rely on a composite network of horizontal and vertical connections that are yet to be fully characterized (Nesper and Fawzi, 2018).

### Structure and Regulation of Blood Flow

The structure of retinal arteries, capillaries and veins is characteristic of a vasculature almost entirely autoregulated for local tissue requirements. The retinal vasculature is deprived of autonomic innervation (Hogan and Feeney, 1963a,b; Laties and Jacobowitz, 1966). Blood flow and local tissue perfusion are adjusted to changes in neuronal activity through myogenic response that involves vasoconstriction and vasodilation in arteries and capillaries (Shepro and Morel, 1993; Haefliger et al., 1994; Lombard, 2006; Peppiatt et al., 2006). Retinal vessels can also adapt blood flow rates to changes in partial pressures of oxygen and carbon dioxide and to variations in the concentration of various molecules essential to retinal metabolism (Pournaras et al., 2008; Aalkjær et al., 2011; Levick, 2018; Yu et al., 2019). Autoregulation is mediated by the endothelium of retinal vessels and by pericytes and smooth muscle cells encompassing them. In contrast with other organs, retinal arteries lack an elastic lamina and the coat of smooth muscle cells enclosing them is more developed. Near the optic disk, this coat comprises five to seven layers of cells. This number decreases to two or three at the equator and to one or two at the periphery (Hogan and Feeney, 1963a; Hogan, 1971). Glial and Müller cells generally lie at the interface between the broad basement membrane enclosing retinal arteries and the adjacent nerve fiber layer. The size and distribution of pericytes surrounding veins is similar to that of smooth muscle cells along arteries (Hogan and Feeney, 1963a; Hogan, 1971). When compared to other tissues, pericytes enclosing retinal capillaries are more numerous and closely spaced (there is approximately a 1:1 ratio between pericytes and endothelial cells). Their basement membrane is adjacent to those of Müller and other glial cells (Hogan and Feeney, 1963b,c; Hogan, 1971).

### Components of the Inner Blood-Retinal Barrier

The continuous endothelium of retinal vessels constitutes the main component of the iBRB. Transfers across this selective barrier occur through passive, carrier-mediated or receptor-mediated transports (see Figure 3). The wall of retinal capillaries is composed of a single layer of endothelial cells enclosed by intramural pericytes and a basement membrane (see Figure 2). A basal lamina separates endothelial cells from pericytes. Passive transport across vessel walls is modulated by the structure and thickness of the basement membrane of capillaries and pericytes, which is thicker than in other organs, and by tight junctions between endothelial cells, which are numerous and extensive (Hogan and Feeney, 1963b; Shakib and Cunha-Vaz, 1966; Hogan, 1971; Frank et al., 1990). The structure of these junctions restricts paracellular transport considerably, so that metabolism substrates and amino acids required for retinal metabolism cross the endothelium predominantly through the transcellular pathway. The permeability of the iBRB to many substrates is known and well-documented (Hosoya and Tachikawa, 2012). Limited evidence suggests that pericytes and smooth muscle cells modulate the molecular permeability of retinal vessels through paracellular transport (Frey et al., 1991). It is unclear if astrocytes and Müller cells impact on the permeability of the iBRB in adults (Vecino et al., 2016). Müller cells may modulate the barrier properties of retinal endothelial cells through the secretion of factors contributing to the formation and maintenance of tight junctions (Tout et al., 1993; Abukawa et al., 2009). In addition, both Müller cells and astrocytes produce many extracellular matrix components including collagens, vitronectin and fibronectin that are likely to impact on mass transfers within the retina (Behzadian et al., 2001).

### Tissue-Level Models of Retinal Perfusion

Models linking retinal blood flow and mass exchange to retinal structures or metabolism at the tissue level have mostly been limited to the study of oxygen delivery and consumption. One of the most basic approaches relies on the Krogh cylinder model, which assumes that oxygen is supplied to a cylindrical region surrounding evenly spaced capillaries (Krogh, 1919; Goldman, 2008). This model predicts the concentration of a passively transported molecule in tissue as a function of its consumption (assumed to be constant), its diffusivity and the radius of the cylinder within which it is entirely consumed. While extended to account for a range of complex processes involved in the delivery of oxygen to the inner retina – including facilitated transport (McGuire and Secomb, 2001), tissue metabolism and time-varying concentrations (Friedland, 1978); the Krogh model is not adapted to model supply regions containing multiple capillaries (Wang and Bassingthwaighte, 2001). Alternative approaches, mainly based on Green’s function (Secomb et al., 2004; Secomb, 2016), have been developed to model oxygen transport in retinal tissue while accounting for non-uniformities in the retinal vascular network and interactions between capillaries (Causin et al., 2016). While built on general principles, these models require a detailed map of the morphology of the retinal vasculature to generate predictions. These maps may be reconstructed from images of the retina (Fry et al., 2018), or generated randomly by harnessing the regularity and fractal nature of the retinal vascular tree (Causin et al., 2016). Because aspects of the retinal vascular network (such as the pattern of connections between different plexuses) are yet to be fully characterized (see Section “Retinal Vascular Pattern”), methods relying on the fractal nature of the retinal vascular tree are generally only relevant to the description of the salient features of the blood flow.

Oxygenation and metabolism in the retina have been experimentally investigated using oxygen-sensitive electrodes and retinal oximetry (Yu and Cringle, 2001; Linsenmeier and Zhang, 2017). These approaches have been applied to explore the resilience of the retina to perturbations including hypoxia and hyperoxia. The main limitation of these approaches is that measurements are often uncoupled from changes occurring at the level of the retinal vasculature. Data collected mainly from animals are at the basis of several mathematical models of oxygen diffusion across the retina (Haugh et al., 1990; Linsenmeier and Padnick-Silver, 2000; Verticchio Vercellin et al., 2021), some of which include considerations on retinal blood flow (Causin et al., 2016).

## Delivery System to Outer Retinal Neurons

The metabolite delivery system to the outer retina has three main components: the choriocapillaris, which is the vascular bed of the choroidal vasculature, Bruch’s membrane and the RPE. These three intrinsically multifunctional compartments are interdependent and compose the outer blood-retinal barrier (see Figure 3).

### Choroidal Vasculature

The choroidal vasculature emerges from lateral, medial, superior and long posterior ciliary arteries (PCA) that arise from the ophthalmic artery (Hayreh, 1962). Each of them divides into to 10 to 20 short posterior ciliary arteries, which cross the sclera near the optic nerve (Wybar, 1954; Hayreh, 1962, 1974c; Hogan, 1971; Virdi and Hayreh, 1987) and supply a distinct sector of the choroid (Hayreh, 1974a,^1975^). They further divide, each subdivision supplying a smaller segment of the choroid. At the end of this vascular tree, arterioles supply the choriocapillaris, an 8–20 μm thick planar capillary bed extending from the optic nerve to the lateral border of the peripheral retina (the ora serrata) (Zouache et al., 2016a). Functionally, choroidal arteries, arterioles, venules and veins form a segmented vascular tree (Hayreh, 1974a,^1975^). The choroid is drained by four vortex veins (one per quadrant), which branch into either the superior or inferior ophthalmic vein (Hayreh, 1990; Oyster, 1999).

The structure of choroidal vessels differs markedly from those forming the retinal vasculature. Smooth muscle cells and pericytes are present along choroidal arteries and veins, respectively; however, their function and subtypes remain poorly characterized in man (Salzman, 1912; Wolter, 1956; Hogan and Feeney, 1961; Hogan, 1971; Condren et al., 2013). Pericytes are the only perivascular cells found in the choriocapillaris. They are horizontally and sparsely distributed, with only 11% of ensheathment observed in adults (compared to 94% in retinal capillaries) (Frank et al., 1990; Chan-Ling et al., 2011). Their function remains unclear (Wolter, 1956; Condren et al., 2013). In contrast with the retinal vasculature, choroidal blood flow sees little to no autoregulation under normal conditions (Bill, 1962; Friedman, 1970; Armaly and Araki, 1975; Bill et al., 1983; Gherezguiher et al., 1991). The presence of extrinsic regulation mediated by sympathetic innervation has been demonstrated in animals including primates (Reiner et al., 2018). This regulation may cause vasoconstriction of arteries and pre-capillary arterioles, which drives the redistribution of arterial blood in the event of an increase in blood pressure (during exercise for instance) (Levick, 2018). Evidence of local regulation of choroidal blood flow mediated through myogenic mechanisms has been found in rabbits (Kiel and Shepherd, 1992; Kiel, 1994; Kiel and van Heuven, 1995).

### The Choriocapillaris

The large diameter of choriocapillaris vessels (Hogan, 1971; Olver, 1990; Ramrattan et al., 1994) demonstrates a weaker resistance to blood flow as compared to retinal capillaries. High blood flow – choriocapillaris blood flow is, per unit mass, three to four times higher than that in the kidney (Weiter et al., 1973) – ensures that gradients of concentrations between the choroid and retina remain steep, and therefore that rates of transfers between these two compartments are maintained high. In addition to low resistance to blood flow, the choriocapillaris features many structures facilitating the movement of molecules across its endothelium. Choriocapillaris vessels are composed of a single layer of endothelial cells enclosed by a basement membrane (Missotten, 1962) and are separated by discontinuous tight junctions. Gap junctions are also observed in the plasma membrane of the endothelium (Hogan, 1971; Spitznas and Reale, 1975), generally on the scleral side of the capillaries and between pericytes and endothelial cells (Spitznas and Reale, 1975). As in glomerular capillaries (Satchell and Braet, 2009), blood vessels in the choriocapillaris contain fenestrations (Hogan and Feeney, 1961; Bernstein and Hollenberg, 1965; Hogan, 1971; Torczynski, 1982). These 600 to 800Å pores spanning the width of the endothelium present with a diaphragm covering part of their surface (Missotten, 1962; Garron, 1963; Hogan, 1971; Spitznas and Reale, 1975), and are more frequent on the retinal side of capillaries (Hogan and Feeney, 1961; Bernstein and Hollenberg, 1965; Federman, 1982).

Whereas fenestrations enhance the diffusion of molecules of small to moderate size (typically with a Euler-Einstein radius of 30–40Å or less), they do not allow for the free transport of macromolecules from plasma to the extravascular space as is often assumed (Nakanishi et al., 2016). In fact, the choriocapillaris substantially restricts the passage of large unreactive molecules (Pino and Essner, 1980, 1981; Törnquist et al., 1990; Grebe et al., 2019) and features receptor-mediated types of transport (Bernstein and Hollenberg, 1965; Pino and Essner, 1981; Nakanishi et al., 2016). Choriocapillaris endothelial cells differentially express several transendothelial transport genes including *CAV1* (caveolin), *TSPO* (cholesterol) and *TFRC* (transferring receptor) (Voigt et al., 2019a). One of the genes most strongly differentially expressed by these cells is plasmalemmal vesicle-associated protein (*PLVAP*). The Plvap protein is present in fenestrations, caveolae and transendothelial channels, and therefore plays a key role in the regulation of transendothelial transport (Wisniewska-Kruk et al., 2016; Bosma et al., 2018; Brinks et al., 2021). Our understanding of the mechanisms and dynamics of the transport of specific macromolecules across the choriocapillaris is limited, and mainly comes from examinations of the movement of albumin. This macromolecule was experimentally found to cross choriocapillaris endothelial cells through caveolae-mediated transcytosis, with an estimated travel time of 30 min or less (Nakanishi et al., 2016).

### Bruch’s Membrane

Molecules reaching the choroidal extravascular space must cross Bruch’s membrane, which occupies the space between the choriocapillaris and the RPE, to reach photoreceptor outer segments (see Figure 3A). Bruch’s membrane forms a selective barrier to the reciprocal transport of molecules between the RPE and choriocapillaris, restricts cellular movement between choroid and retina and physically supports RPE adhesion (Booij et al., 2010). Histologically part of the choroid, Bruch’s membrane is 2 to 5 μm thick and is composed of an elastin layer sandwiched by two layers of collagen fibers (the inner and outer collagenous layers). Its inner- and outermost layers are basement membranes to the RPE and choriocapillaris, respectively (Hogan, 1961). The inner and outer collagenous layers are composed of various forms of collagen arranged in a grid-like structure and embedded in a mass of interacting biomolecules. The elastin layer is made up of coarse interlaced linear elastin fibers extending from the optic nerve to the far retinal periphery. The outer basement membrane of Bruch’s membrane is composed of several forms of collagen. Because it coincides with the basement membrane of the choriocapillaris, it is discontinuous – it is present close to capillaries of the choriocapillaris but absent in the space separating them (Marmor and Wolfensberger, 1998).

Since Bruch’s membrane is acellular, transport across it is passive and entirely determined by its thickness and molecular composition. Gradients of hydrostatic pressure and concentration drive the movement of molecules, which either cross the membrane or bind to it (Marmor and Wolfensberger, 1998; Strauss, 2005). The average diameter of gaps within the elastin layer and its thickness are important determinants of the diffusive properties of Bruch’s membrane (Chong et al., 2005). Out of the five layers composing it, the inner collagenous layer has been experimentally found to form the main resistance to fluid movement, and is therefore the principal determinant of Bruch’s membrane’s hydraulic conductivity (Starita et al., 1997). Controversy exists regarding the maximal size of molecules able to travel across Bruch’s membrane. Intravenous injections of protein tracers in animals indicate that particles 64–500 kDa in weight enter Bruch’s membrane but do not cross it (Bernstein and Hollenberg, 1965; Pino and Essner, 1981; Pino and Thouron, 1983). However, molecules 40–200 kDA have been shown to cross the membrane *in vitro* (Moore and Clover, 2001). This discrepancy is likely to be caused by the experimental systems used in *in vitro* studies, which may not replicate physiologically realistic gradients of pressure and normal fluid fluxes, and often involve both advective and diffusive transports across the membrane (Hussain et al., 2010).

### Retinal Pigment Epithelium

At the level of the RPE, tight junctions connecting adjacent cells ensure that the outer blood-retinal barrier is maintained (Raviola, 1977). The resistance to paracellular transport across the RPE was estimated to be ten times higher than that to transcellular transport in animals (Miller and Steinberg, 1977a,b). Choriocapillaris fenestrations are maintained by growth factors secreted by the RPE that diffuse across Bruch’s membrane (Blaauwgeers et al., 1999; Kamba et al., 2006; Kim et al., 2019), highlighting the close relationship between these three components. The expression of *PLVAP* may be induced and regulated by growth factors produced at the level of the RPE including vascular endothelial growth factors (Vegf) (Marneros et al., 2005; Bosma et al., 2018) and pigment epithelium-derived factor (Pedf) (Farnoodian et al., 2018). Vegf exposure has been shown to alter the vascular permeability of endothelial cells (Bates, 2010), partly by modulating the frequency and structure of their fenestrations (Stan, 2007).

### Tissue-Level Models of Exchange Between Choroid and Retina

Tissue-level analyses of the movement of molecules between retina and choriocapillaris, which describe the combination of phenomena occurring at the level of choroidal endothelial cells, Bruch’s membrane and the RPE, are scarce. Salient features of the transfer of small passively transported molecules may be inferred from theoretical and experimental analyses of oxygen concentration profiles (Linsenmeier and Zhang, 2017; Yu et al., 2019). These studies indicate that passively transported molecules diffuse along a concentration gradient that is perpendicular to the plane of the choriocapillaris. In the case of oxygen, this gradient is directed toward the outer retina.

Blood enters and leaves the choriocapillaris through a set of arterioles and venules connected to the surface furthest from the retina approximately perpendicularly to the plane of the capillaries. Because of this arrangement, the blood flow is decomposed into contiguous functional vascular segments separated by separation surfaces across which there is no flow (Zouache et al., 2016a). Groups of functional vascular segments are commonly referred to as lobules (Hayreh, 1974b), a term introduced by analogy with liver functional units (Torczynski and Tso, 1976). The pattern of segmentation of the choriocapillaris blood flow is determined by pressure gradients between arteriolar and venular insertions and their relative distribution (Flower et al., 1995; Zouache et al., 2016a). The extraction of diffusive species from the choriocapillaris and the distance across which they diffuse across the retinal tissue is determined by the velocity of blood flowing through the choriocapillaris and by the relative distribution and flow rate of arterioles and venules inserting into its plane. Blood velocities across the choriocapillaris have been experimentally and theoretically shown to be spatially heterogeneous (Flower, 1993; Flower et al., 1995; Zouache et al., 2016a). Mass exchange between choriocapillaris and outer retina (denoted η) is at the level of a functional vascular segment (basic lobule unit) described by the theoretical relation:


$$\eta =\frac{A_{0}\,\text{h}}{\tau \,Q_{a}}\,\text{Φ}$$

where *A*_*0*_ is the surface area of the lobule, *h* the local thickness of the choriocapillaris, *Q*_*a*_ is the flow rate in the arteriole feeding the lobule, Φ is the vascular volume fraction of the choriocapillaris (the volume of tissue occupied by capillaries) and 1/τ is a transfer coefficient specific to the compound considered (Zouache et al., 2015, 2016a). This relation may also be expressed as:


$$\eta =\frac{<T>}{\tau }$$

where < *T* > is the mean travel time of blood between an arteriole and a venule supplying and draining a choriocapillaris lobule, respectively. Both relations are functions of the ratio of arterioles and venules supplying functional vascular segments. Based on experimental data generated in animals, the extraction rate of glucose and oxygen is between less than one percent and five percent per volume of blood (Alm and Bill, 1972; Wang et al., 1997; Linsenmeier and Padnick-Silver, 2000), which yields τ ≈ 0.2−1×10^5^*s*. In the case of glucose, this estimate is partly determined by the abundance of GLUT receptors on the plasma membrane of choroidal endothelial cells (Mantych et al., 1993), RPE (Strauss, 2005) and photoreceptor outer segments (Hsu and Molday, 1991).

## Spatial Variations in Neuron Populations and Inner and Outer Retinal Mass Transport Systems

**FIGURE 4:**
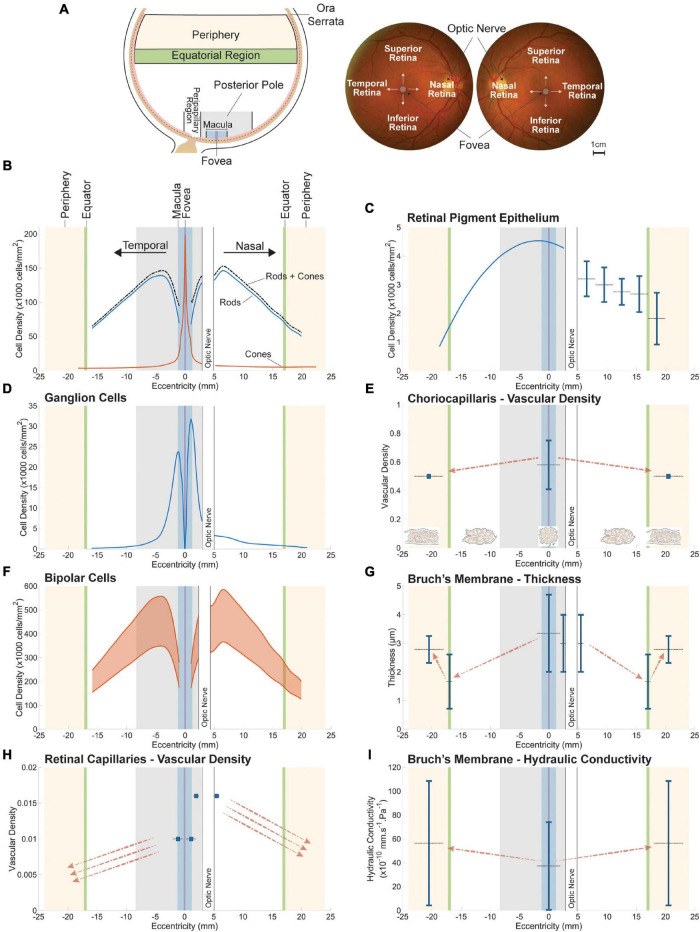
Spatial variations in retinal neuron populations **(B,D,F)** and in the key structures of their respective metabolite delivery systems **(C,E,G–I)**. The approximate location of sampled retinal regions and their associated denomination is described in **(A)**. Terminologies vary between studies; the ones employed here are consistent with the main text. Plotted data were collected from publications listed in Supplementary Table 1. All spatial variations are displayed along the nasal-temporal axis as illustrated in **(B)**. Reported values are plotted as vertical line segments (ranges) or single points, and the approximate region they apply to is delineated using horizontal dashed lines. Dashed arrows indicate qualitatively reported trends. The density of bipolar cells **(F)** is plotted as a range inferred from approximate ratios between their density and that of their respective photoreceptor type. The maximal vascular density of retinal capillaries **(H)** appears to overlap with the highest density of bipolar **(F)** and ganglion **(D)** cells. Bruch’s membrane is thickest at the fovea **(G)**, where photoreceptor density **(B)** and choriocapillaris vascular density **(E)** are highest.

In this section, we explore the relation between retinal neuron populations and their respective mass transport systems by harnessing spatial variations in the structure of the retina and choroid. This variability evolved as an adaptation to spatial variations in light intensity, contrast and amplitude of visual aberrations across the retina (Walls, 1942; Hughes, 1977). To optimize spatial resolution and sampling of light (Hirsch and Hylton, 1984; Hirsch and Curcio, 1989), the size, density, and tilling geometry of photoreceptors vary across the eye. In addition, as in many vertebrates (Walls, 1937, 1942), the human eye contains a region dedicated to high resolution vision, the *fovea centralis*, which allows for the capture a small part of retinal images only but in great details (Hughes, 1977). Anatomically, the fovea consists of a 1500 μm-wide depression in the retinal tissue caused by the absence of inner retinal layers; see Figure 1C. In this region the retina is only 100 μm thick, and is deprived of the inner nuclear, inner plexiform, ganglion cell and nerve fiber layers as well as retinal capillaries (Hogan, 1971). The spatial variation in the human photoreceptor mosaic can also be seen in other cells and structures of the retina and choroid, including ganglion cells and retinal and choroidal vasculatures. The location and classical denomination of retinal regions are described in Figure 4A.

### Retinal Neurons

Methods based on histology of human donor eyes (Osterberg, 1935; Polyak, 1941; Farber et al., 1985; Curcio et al., 1987, 1990; Jonas et al., 1992) and *in vivo* imaging techniques (Chui et al., 2008a,b; Song et al., 2011; Zhang et al., 2015) have shown that the density of cones is maximal at the fovea (see Figure 4B). Its reported peak can vary by several orders of magnitude between individuals, ranging from 49,600 (Dorey et al., 1989) to 238,000 (Ahnelt et al., 1987) cones/mm^2^ on average. Inter-individual variability varies with location in the retina (Zhang et al., 2015; Elsner et al., 2017) and may be partly explained by differences in axial length between eyes sampled (Chui et al., 2008a; Legras et al., 2018). The most detailed sampling of the cone mosaic in human donor eyes to date reported a mean peak density of 199,000 cones/mm^2^ at the foveal center (Curcio et al., 1987, 1990), which is consistent with measurements made using AO (Zhang et al., 2015). Cone density decreases sharply with distance from the center of the fovea (Osterberg, 1935; Curcio et al., 1987, 1990; Jonas et al., 1992; Chui et al., 2008a; Song et al., 2011; Zhang et al., 2015), and is 40–45% higher in the nasal retina as compared to the temporal region (Curcio et al., 1990). It is also slightly larger in the midperipheral inferior region of the fundus as compared to the superior retina (Osterberg, 1935; Curcio et al., 1990; Jonas et al., 1992). Certain cone subtypes have different distributions, which do not appear to be either random or regular (Curcio et al., 1991; Mollon and Bowmaker, 1992; Roorda and Williams, 1999). Rods are absent from the fovea (Osterberg, 1935; Polyak, 1941; Farber et al., 1985; Curcio et al., 1987, 1990; Jonas et al., 1992). The diameter of the region of the fovea deprived of rods is 0.35 mm on average (Curcio et al., 1990). The density of rods is largest in the nasal region of the retina (Osterberg, 1935; Curcio et al., 1990; Jonas et al., 1992), with a maximal density found in the vicinity of the optic disk (approximately 3–5 mm from the foveal center) (Curcio et al., 1990; Jonas et al., 1992). Peak rod density ranges from 135,000 (Farber et al., 1985) to 176,000 rods/mm^2^ (Curcio et al., 1990) in this region. Differences in rod density between individuals can reach 10%, and are much smaller than those observed for cones (Curcio et al., 1990). In addition to their density, the morphology of photoreceptors varies with location in the eye. The diameter of cones increases from approximately 1.6 (Curcio et al., 1990) to 2.23 μm (Scoles et al., 2014) at the center of the fovea to 8–10 μm in the periphery of the retina (Curcio et al., 1990; Jonas et al., 1992; Scoles et al., 2014). The diameter of rods increases from approximately 3 μm in the region with the highest rod density to 5.5 μm in the peripheral retina (Curcio et al., 1990; Jonas et al., 1992).

The ratio between photoreceptors and bipolar cells varies across the eye. At the fovea, bipolar cells are connected on average to one cone and one ganglion cell, thus forming a one-to-one wiring. Multiple connections are observed between bipolar cells and photoreceptors in the region extending from outside the fovea to the peripheral retina, which provides pooling of information over space. Ganglion cells in this region connect to multiple bipolar cells and receive signal originating from distinct photoreceptors (Oyster, 1999). Overall, the density of rod and cone bipolar cells follows the distribution of their respective photoreceptor type. The density of cone bipolar cells is 2.5 to 4 times larger than that of cone photoreceptors whereas rod bipolar cell density is approximately a tenth of that of rods (Martin and Grünert, 1992; Grünert et al., 1994); see Figure 4F.

The distribution of horizontal cells has yet to be determined in humans; however, their density has been estimated in monkey retinas. From a minimum of 250 cells/mm^2^ at the foveal center, it increases rapidly at the edge of the fovea. It reaches a maximum of approximately 23,000 cells/mm^2^ within an annulus of 0.6 mm radius enclosing the fovea (Röhrenbeck et al., 1989). This peak is reached in the region where pedicles of foveal cones are displaced (Tsukamoto et al., 1992). Outside of the fovea, the density of horizontal cells decreases approximately exponentially out to the peripheral retina, in keeping with the distribution of cones (Röhrenbeck et al., 1989; Wässle et al., 1989; Oyster, 1999). In the peripheral retina this density is twenty times smaller than the peak observed close to the fovea (Röhrenbeck et al., 1989).

Absent in the fovea, ganglion cells appear approximately 100–500 μm from the foveal center. Their peak density varies greatly between individuals and ranges from 32,000 to 38,000 cells/mm^2^ (Curcio and Allen, 1990), although studies using AO reported lower values (Liu et al., 2017). This maximum is reached within a horizontally oriented elliptical ring located 0.4 to 2.0 mm from the foveal center. Ganglion cell density decreases sharply with distance from the foveal center, reaching approximately 100 cells/mm^2^ in the retinal periphery, and does not correlate with cone density (see Figure 4D). This pattern of variation is not uniform across the eye. At similar distances from the foveal center, the density of ganglion cells in the nasal retina exceeds that of the temporal region by more than 300%. It is in the superior retina larger than in the inferior region by more than 60% (Curcio and Allen, 1990). The mean peak density of melanopsin-expressing retinal ganglion cells decreases from approximately 20–40 cells/mm^2^ at 2 mm from the center of the fovea to 10 cells/mm^2^ at about 8 mm eccentricity (Nasir-Ahmad et al., 2019). The topography of amacrine cells has not been mapped in humans. Evidence from monkeys indicates that their distribution, density and coverage varies between subtypes (Dacey, 1990; Wässle et al., 1995). Their density peaks close to the fovea and declines with distance from the foveal center (Dacey, 1990; Rodieck and Marshak, 1992; Wässle et al., 1995).

Differences in the function, distribution and morphology of retinal cells between fovea and peripheral retina are associated with marked differences in gene expression (Peng et al., 2019; Voigt et al., 2019b,^2021^; Yan et al., 2020). Using unstructured statistical methods that assessed how close transcription profiles from a large number of cells are, foveal cones were found to form clusters that were distinct from their peripheral counterparts (Voigt et al., 2019b). This method also differentiated between distinct cone subtypes (Lukowski et al., 2019).

### Glial Cells

The mean density of Müller cells across the retina in man was estimated to be approximately 11,000 cells/mm^2^, a number that is fairly consistent among mammals (Dreher et al., 1992). Information on spatial variations in the density and morphology of these cells is limited. In the monkey retina, the density of Müller cells varies between 6000 cells/mm^2^ in the periphery and more than 30,000 cells/mm^2^ in the parafoveal region. Müller cells are generally longer in the central retina as compared to the periphery, and the average proximity of neighboring cells increases with distance from the fovea (Distler and Dreher, 1996). The distribution of astrocytes across the monkey retina is uneven. Their concentration, which correlates with the thickness of the nerve fiber layer, is maximal in the proximity of the optic nerve and is particularly low in the perifoveal region. Astrocytes of the nerve fiber layer appear as star-shaped cells in the periphery but seem bipolar close to the optic nerve. The morphology of astrocytes present in the ganglion cell layer is consistent across the retina (Büssow, 1980; Distler et al., 1993).

### Retinal Vasculature

There is no evidence of spatial variation in the structure and ultrastructure of blood vessels across the retina (Pournaras et al., 2008). Blood flow to the temporal part of the retina is greater than that to the nasal region (Riva et al., 1985; Feke et al., 1989); it is also greater in the superior quadrant as compared to the inferior retina (Tomita et al., 2020). These differences are likely to be associated with spatial variations in the perfusion of retinal capillary beds across the retina. The arrangement and number of plexuses composing the retinal capillary network varies spatially. The fovea is deprived of retinal capillaries. Close to the FAZ, retinal capillary plexuses merge into a single layer of capillaries (Campbell et al., 2017; Nesper and Fawzi, 2018). The superficial capillary network is present across most of the retina. The intermediate and deep capillary plexuses may be seen in the macula and posterior pole, but merge into one network peripherally (Toussaint et al., 1961; Hogan, 1971; Campbell et al., 2017). The radial peripapillary plexus is observed in the peripapillary region and in part of the macula (Michaelson, 1956; Kornzweig et al., 1964; Henkind, 1967; Jia et al., 2014; Campbell et al., 2017).

The density of retinal vessels is partly determined by the thickness of the portion of the retina that they supply (Michaelson, 1956; Chase, 1982; Buttery et al., 1991; Snodderly et al., 1992). While the combined volume of retinal vessels in the deeper networks remains constant across the eye, the cumulated vascular volume of superficial retinal vessels increases with the thickness of the nerve fiber layer. The fact that this layer contributes more to vascularity than to retinal thickness indicates that the density of retinal vessels is more likely to be determined by local metabolic requirements (and diffusion distances) than by tissue volume (Snodderly et al., 1992). This observation is further strengthened by the fact that the diameter of capillaries is larger in the superficial nerve fiber layer as compared to the inner nuclear layer (Snodderly et al., 1992; Tan et al., 2012). This indicates a smaller resistance to blood flow in this layer and a higher propensity for passive molecular exchange.

Analyses of human donor eyes indicate that the greatest density of retinal capillaries, expressed as a percentage of total retinal volume, is found at the margin of the avascular fovea, where it reaches approximately 1% (Snodderly et al., 1992); see Figure 4H. The density of capillaries decreases gradually toward the mid-periphery and periphery of the eye. In these regions the retina is thinner and capillaries are fewer in number (Toussaint et al., 1961; Kornzweig et al., 1964; Hogan, 1971). Histological studies (Snodderly et al., 1992) found that the density of capillaries in the vicinity of the optic nerve is approximately 1.6–1.7% of the volume of the retina and decreases with distance from the optic nerve head. Capillary density extracted from OCTA slabs is generally calculated by dividing the surface area of automatically or manually traced capillaries by the area of the retina sampled. Comparisons between densities extracted from histology and OCTA using perfused human donor eyes (An et al., 2018; Balaratnasingam et al., 2018) and animal eyes (Yu et al., 2021) indicate that OCTA provides a good representation of large retinal vessels but does not visualize all retinal capillaries. Estimates of capillary density obtained from OCTA slabs range from 10 to 60% on average in similar regions of the retina (Coscas et al., 2016; Iafe et al., 2016; Garrity et al., 2017; Lavia et al., 2019). In comparison, histological studies indicate that the percentage of retinal area occupied by capillaries lies between 40 and 55% (Snodderly et al., 1992; An et al., 2018). It increases steeply in the parafovea and reaches a maximum approximately 1.5 mm from the foveal center (Snodderly et al., 1992). OCTA studies have found that the density of the deep capillary network is greater than that of the superficial capillary network (Lavia et al., 2019).

### Choroidal Vasculature

The choroidal vasculature displays marked variations in its anatomy and physiology across the eye. The number of arterioles and venules connected to the choriocapillaris per unit of volume is maximal in the submacular region and decreases toward the periphery. The ratio of arteriolar to venular insertions follows a similar pattern, varying from up to 5:1 in the submacular area to 1:4 in the periphery (Amalric, 1983; Fryczkowski et al., 1991; Fryczkowski, 1994). Because of its unconventional morphology and large local variations, quantifying differences in the structure of the choriocapillaris across the eye has proven challenging. Qualitatively, capillaries are narrowest in the posterior pole (Ring and Fujino, 1967; Hogan, 1971; Torczynski, 1982) and become progressively wider in the periphery (Ring and Fujino, 1967; Hogan, 1971). The space between capillaries (called *septa*) follows a similar pattern, varying between about 3 and 18 μm in diameter at the posterior pole (Hogan, 1971) and widening into longer and thinner channel-like structures toward the equator (Salzman, 1912; Wybar, 1954; Hogan, 1971; Torczynski and Tso, 1976); see Figure 4E. Septae further elongate and widen toward the periphery and become loose in the region of the ora serrata (Salzman, 1912; Klien, 1966; Yoneya et al., 1983; Fryczkowski et al., 1991). Variations in the diameter of capillaries and septae across the eye are best described by a measure of vascular density. The vascular density of the choriocapillaris has a strong dependence on age. In the macula, it varies between 0.75 and 0.41 over a lifespan (Ramrattan et al., 1994). Measurements unadjusted for age indicate that the density of the choriocapillaris is approximately 0.5 on average in the peripapillary and peripheral regions. It does appear to decline more abruptly in the periphery (Ramrattan et al., 1994; Spraul et al., 1999, 2002); see Figure 4E. Choriocapillaris density seems to be independent of extrinsic anatomical features such as the thickness of Bruch’s membrane or choroid and correlates with age better than capillary or septae sizes (Ramrattan et al., 1994).

Variations in the anatomy of the choriocapillaris translate important differences in the shape of functional lobules and possibly their mass exchange with the outer retina. These differences are yet to be fully characterized *in vivo*; however, some of them can be inferred from anatomical studies by using existing theoretical frameworks (Zouache et al., 2015, 2016a). Estimates of the average distance (Torczynski and Tso, 1976; Fryczkowski and Sherman, 1988; Olver, 1990) and ratio (Amalric, 1983; Fryczkowski et al., 1991; Fryczkowski, 1994) between arteriolar and venular insertions into the choriocapillaris, of their respective number (Araki, 1976) and variations in vascular density (Ramrattan et al., 1994; Spraul et al., 1999, 2002) indicate that the surface area of lobules is smallest in the submacular area, where their shape is closest to a regular square, pentagon or hexagon. Toward the periphery, the surface of exchange between choriocapillaris and outer retina is larger, and likely to take the shape of an elongated rectangle. Because of a lack of hemodynamic data, it is unclear if differences in the characteristics of functional lobules across the eye are synonymous with spatial variations in transfers between choriocapillaris and outer retina.

Spatial variations in the ultrastructure of choroidal vessels have seldom been examined. Limited data indicate that fenestrations are more frequent in the submacular choriocapillaris as compared to the periphery of the eye (they cover 60.3% of endothelial cell walls in the fovea vs. 36.7% in the periphery) (Federman, 1982).

### Bruch’s Membrane

The thickness and composition of Bruch’s membrane are the main structural factors assessed to characterize its diffusive properties. This thickness appears to be larger in the vicinity of the optic nerve head (Salzman, 1912; Garron, 1963) and in the periphery of the eye (Nakaizumi, 1964; Nakaizumi et al., 1964) as compared to the posterior pole and submacular regions (see Figure 4G). The composition of the elastin layer varies across the retina. Qualitatively, it is thicker and occupies a larger portion of Bruch’s membrane in the posterior pole (Garron, 1963) and submacular area (Chong et al., 2005) as compared to the peripapillary region; although a large variability is observed between samples. Spatial variations in the porosity of Bruch’s membrane were assessed quantitatively by measuring the integrity of this layer, defined as the total length of detectable elastin divided by the length of the portion of membrane considered. This measure is correlated with the thickness of the elastin layer. Elastin integrity is minimal at the fovea (approximately 40%). It is on average 60% higher in the periphery as compared to the subfoveal choroid (Chong et al., 2005). *In vitro* experiments indicate that the hydraulic conductivity of Bruch’s membrane is higher in the retinal periphery as compared to the submacular region (Moore et al., 1995); see Figure 4I.

### Retinal Pigment Epithelium

The density of RPE cells is maximal at the fovea and decreases almost linearly with distance from the foveal center (Ts’o and Friedman, 1968; Streeten, 1969; Young, 1971; Gao and Hollyfield, 1992; Harman et al., 1997; Bhatia et al., 2016; Granger et al., 2018; see Figure 4C); although a large variability is observed between subjects. A decrease in the density of RPE cells is associated with an increase in their mean surface area (Ts’o and Friedman, 1968; Granger et al., 2018) and a reduction in the average number of neighbors to each cell (Bhatia et al., 2016). Hexagonal cells are significantly more frequent in the macula than in the peripheral retina (Watzke et al., 1993). The mean cone-to-RPE cell ratio is maximal at the fovea and decreases rapidly starting from 2 mm from the foveal center (Granger et al., 2018).

### Conclusion

Most investigations reporting spatial variations among retinal neuron populations, retinal vasculature and choroid have considered these systems in isolation. In part because of this, combining published measurements is not sufficient to capture local differences in retinal and choroidal structures that may indicate a correlated pattern of variation. This “lack of resolution” is exacerbated by variabilities in regions of the retina sampled between studies. Therefore, similarities between the pattern of change in retinal and choroidal structures across the eye can only be described qualitatively.

Past investigations indicate that spatial variations in the structure and physiology of retinal circulatory systems follow those observed among the populations of cells that they support. The density of retinal capillaries (expressed as a function of retinal volume) is maximal close to the optic nerve head, where the concentration of ganglion and bipolar cells is highest. It decreases toward the periphery, where the density of retinal neurons is comparatively reduced. The amplitude of differences in vascular density displayed by the choriocapillaris across the choroid seem almost marginal when compared to those seen among photoreceptors. Variations in the thickness and hydraulic conductivity of Bruch’s membrane indicate that this structure forms a greater impediment to mass exchange in the submacular area as compared to the periphery. Therefore, barriers to transport appear to be more selective in regions where the density of photoreceptors is highest.

## Relation Between Neurons and Retinal Mass Transport Systems in Aging

**FIGURE 5:**
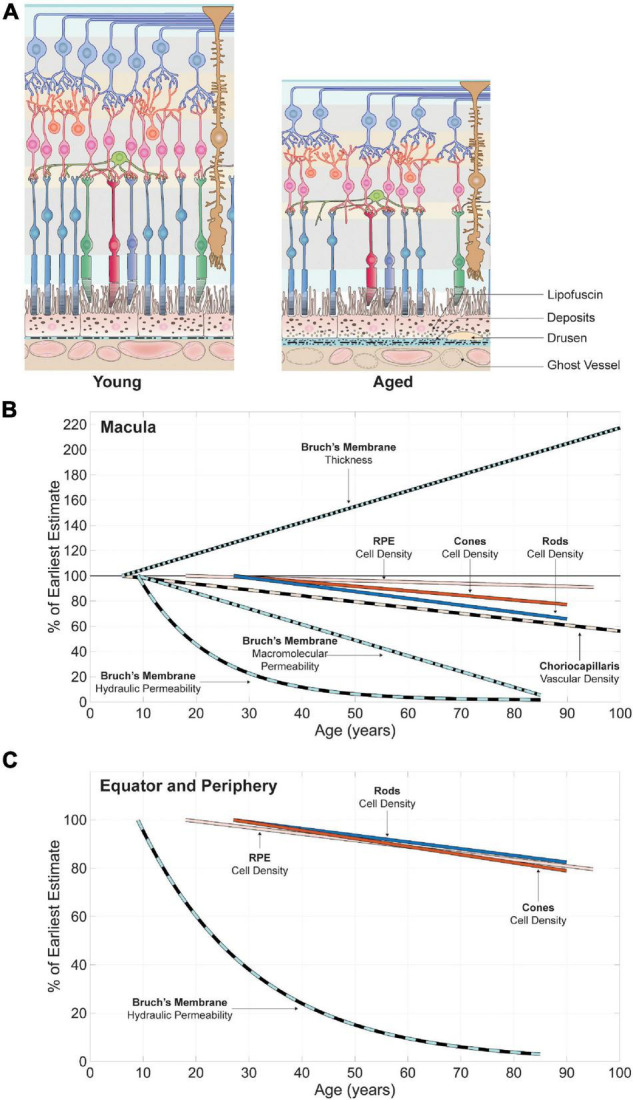
Age-related variations in retinal neuron populations and in the key structures of their respective metabolite delivery systems in the macula and equator and periphery. A schematic of changes affecting the retina and choroid adapted and modified with permission from Campello et al. (2021) is shown in **(A)**. Linear and non-linear regressions were collected from publications listed in Supplementary Table 2. The features plotted are normalized by the first value of each regression, which corresponds to the estimate in the youngest eye included in each study. In the macula **(B)**, there can be large inter-individual differences that mask variations in the density of RPE cells and cone photoreceptors with age. With careful elimination of disease and other extraneous variables, some older subjects have significantly lower cone densities than do younger subjects. Aging is associated with an increase in the thickness of Bruch’s membrane and a reduction in its hydraulic and macromolecular permeability. The rate of rod loss and the decrease in the vascular density of the choriocapillaris observed with age lie within a similar range. In the equatorial and peripheral region **(C)** loss of cones with age is more pronounced as compared to rods.

Aging can provide valuable insights into the relation between retinal neuron populations and mass transport systems, including information about its resilience to perturbations occurring over large timescales. Many structural changes have been observed in the aging retina (see Figure 5A). Some reports are conflicting, partly because the effect of aging on retinal cells and structures varies across the eye. Inconsistencies or inaccuracies in the regions of the retina sampled may therefore introduce large variabilities in quantified features, rendering any generalization difficult. In addition, the ability to detect cellular changes or losses caused by aging are limited by large inter-subject variabilities (Gao and Hollyfield, 1992) present at baseline, as seen in Section “Spatial Variations in Neuron Populations and Inner and Outer Retinal Mass Transport Systems,” and by differences in the range of ages considered in each study. Finally, it is often difficult to determine if earlier assessments of retinal aging considered samples presenting features that would today be classified as pathological. The definition of many vitreoretinal diseases has evolved considerably over the years, and several phenotypes previously considered as age-related are now known to be associated with degenerative diseases. For instance, changes caused by age-related macular degeneration (AMD) often overlap with those associated with aging prior to the onset of clinical symptoms. However, the existence of genetic associations with AMD (Fritsche et al., 2016) and disease specific phenotypes (Ardeljan and Chan, 2013) indicates that the chain of events associated with the onset and progression of this disease has components that are independent of the aging process.

### Foveal and Macular Regions

#### Inner Retina

While this is disputed (Oyster, 1999; Harman et al., 2000) due to methodological inconsistencies (Calkins, 2013), cells in the ganglion cell layers may be lost in aging (Gao and Hollyfield, 1992; Curcio and Drucker, 1993). Their density was estimated to decrease by 16% on average between the second and sixth decade (Gao and Hollyfield, 1992). Differences between young and old eyes appear to be more pronounced approximately 3 mm from the foveal center, where losses in mean density reach 25% (Curcio and Drucker, 1993). Data on horizontal, bipolar and amacrine cells are scarce. It is generally assumed that their density remains constant throughout adulthood (Oyster, 1999). Limited data do point to a 21 to 27% decline in bipolar cell density with age over years spanning from the third to the seventh decade (Aggarwal et al., 2007); however, the region of the retina where measurements were made was not specified. Endothelial degeneration (Nag and Wadhwa, 2012) and acellularity of single vessels (Kuwabara et al., 1961), sometimes associated with loss of mural cells, are often seen among retinal capillaries. Acellularity is however not necessarily significative of functional abnormalities or structural changes in adjacent parts of the retina (Kuwabara and Cogan, 1965). A gradual reduction of macular retinal thickness is often observed with age, generally after the fourth decade (Eriksson and Alm, 2009; Karampelas et al., 2013; von Hanno et al., 2017; Ryoo et al., 2018; Zouache et al., 2020; Trinh et al., 2021). The thickness of the ganglion cell, inner plexiform, inner nuclear and outer nuclear layers demonstrate similar rates of decline with age. In contrast, the thickness of the nerve fiber layer does not vary significantly with age (Trinh et al., 2021). *In vivo* methods have found that the vascular density of deep and superficial retinal capillary layers decreases by 0.55–0.86% per decade on average (Wei et al., 2017; Lavia et al., 2019). This variation is associated with a reduction of the mean blood flow velocity among retinal venules (Wei et al., 2017) and a decrease in retinal tissue perfusion (defined as the blood flow supplying the macula divided by the sampled volume of the inner retina) (Lin et al., 2019). The effect of perturbations of the retinal blood flow on metabolism has been investigated experimentally (Linsenmeier and Zhang, 2017; Yu et al., 2019); however, none of these perturbations occurred over a timescale relevant to the aging process.

#### Outer Retina and Choroid

The amplitude of changes observed in the outer retina and choroid appears larger in magnitude than those documented in the inner retina. Displacement and patchy loss of nuclei in cone outer segments have been observed in the macular retina (Gartner and Henkind, 1981; Dorey et al., 1989; Gao and Hollyfield, 1992). Analyses of human donor eyes suggest that the density of foveal cones decreases with age, although this reduction was not found to be statistically significant (Gao and Hollyfield, 1992; Curcio et al., 1993). Assessments of variations in cone density with age may however be affected by axial length (Park et al., 2013; Wang et al., 2019), undiagnosed retinal pathology and discrepancies in regions sampled (Elsner et al., 2017), which AO-based techniques can control for. Studies using AO have found that older eyes have significantly lower cone densities as compared to younger ones in regions 500 μm to 4 mm (Chui et al., 2012) and 570 to 580 μm (2°eccentricity) (Legras et al., 2018) from the foveal center. Differences in cone density between younger and older subjects are largest near the fovea and decrease with distance from its center (Song et al., 2011; Chui et al., 2012). Loss of rod photoreceptors is observed in the region inferior to the fovea. Starting at the fifth decade, the proportion of rods lost reaches 30% by the ninth decade within an annulus ranging from 0.5 to 3 mm from the foveal center (see Figure 5B). Interestingly, this annulus of greatest rod loss lies in a region distinct from the part of the retina displaying the highest rod density. The drop in rod density does not result in reduced rod coverage because the space vacated by lost cells is filled by larger rods (Curcio et al., 1993), thereby maintaining their overall tiling (Oyster, 1999). Loss of RPE at the foveal center (Gao and Hollyfield, 1992) and in the macular and paramacular retina (Ts’o and Friedman, 1968; Dorey et al., 1989) with age have been reported; although this finding has been disputed (Watzke et al., 1993; Harman et al., 1997). Reported variations in cell density may in fact be caused by an increase in the surface area of the retina with age (Harman et al., 1997). An increase in the mean surface area of RPE cells and a reduction of the frequency of hexagonal cells (Watzke et al., 1993; Bhatia et al., 2016) have been observed in aging. These morphological changes are likely to preserve the continuity of the RPE layer, which may be affected by cell loss or changes in retinal area. Some have reported that the ratio between cone and RPE cells lies within a similar range across all age groups (Gao and Hollyfield, 1992). The decrease in retinal thickness with age after the fourth decade (Eriksson and Alm, 2009; Karampelas et al., 2013; von Hanno et al., 2017; Ryoo et al., 2018; Zouache et al., 2020; Trinh et al., 2021) is not attributable to the thickness of the outer plexiform or RPE layers, which do not vary significantly with age (Trinh et al., 2021).

Analyses of OCT volume scans suggest that the total choroidal and mean choroidal luminal areas decrease with age (Nivison-Smith et al., 2020). Choroidal blood flow and volume, measured using laser Doppler flowmetry and expressed in arbitrary units, have been experimentally found to decrease by 7.5 and 8.8% per decade, respectively (Grunwald et al., 1998). Investigations of variations in choroidal blood velocities with age have yielded conflicting results (Grunwald et al., 1998; Straubhaar et al., 2000). The mean cross-sectional diameter of vessels forming the choriocapillaris (equivalent to choriocapillaris thickness) and its vascular density decline by up to 45 and 34% between the first and tenth decade, respectively (Ramrattan et al., 1994). This latter finding may be correlated with the increase in flow deficits observed *in vivo* using OCTA (Zheng et al., 2019). The effect of these changes on the transfer of molecules to the outer retina is unclear. Theoretical considerations suggest that a reduction in choroidal blood flow may result in smaller blood velocities within the choriocapillaris, causing an increase in spatial heterogeneities in mass transfers between this vascular bed and the outer retina (Zouache et al., 2016c). Mathematical models indicate that reduced choriocapillaris thickness causes an increase in resistance to blood flow (Zouache et al., 2015), but is also synonymous with larger passive transfer rates between the choriocapillaris and the outer retina (Zouache et al., 2019). A reduction in vascular density results in a decline in the surface area of the choriocapillaris available for exchange; however, it may also have a positive effect on rates of transfers to the outer retina by causing a local increase in blood velocity. The significance of the observed decline in submacular total choroidal thickness with age (Wakatsuki et al., 2015) is yet to be understood. Increasing age is associated with a thickening of Bruch’s membrane by a proportion reaching 135% between the first and tenth decade on average (Ramrattan et al., 1994), a loss of definition of the elastin layer and an accumulation of matrix and non-matrix material rich in lipids within its sublayers (Hogan, 1967; Feeney-Burns and Ellersieck, 1985; Pauleikhoff et al., 1990; Holz et al., 1994; Karwatowski et al., 1995; Johnson et al., 2007). Increased cross-linking of fibers and accumulations of advanced glycation end products (Karwatowski et al., 1995; Handa et al., 1999) are also observed. These structural changes begin early in life (Feeney-Burns and Ellersieck, 1985) and alter the porosity and diffusive properties of the membrane in a molecule-specific manner. The hydraulic conductivity of Bruch’s membrane decreases exponentially with age, being halved every 9.5 years on average (Moore et al., 1995). This variation is partly explained by the accumulation of extractable lipids within the membrane (Curcio et al., 2011), which follows a reciprocal pattern (Holz et al., 1994). *In vitro* experiments have demonstrated a 45–65% linear decrease of the transport of amino-acids across Bruch’s membrane over a lifespan (Hussain et al., 2002). The diffusional transport of macromolecules (such as dextran) accross the membrane is reduced by 93.5% between the first and ninth decade (Hussain et al., 2010). The maximal size of serum proteins crossing Bruch’s membrane is progressively reduced from approximately 200 kDa in the first decade to 100 kDa in the ninth decade, and is associated with a 10-fold reduction in their transport over this time (Moore and Clover, 2001).

### Equatorial and Peripheral Regions

At the temporal equator, the density of cones is reduced by 23% on average by the ninth decade (Curcio et al., 1993); see Figure 5C. The average rate of decrease of cones and RPE cells is uniform and estimated to be approximately to 16 and 14 cells/mm^2^/year, respectively (Gao and Hollyfield, 1992). Some have reported stable rod counts in this region throughout adulthood (Curcio et al., 1993) whereas others observed non-uniform rates of decrease with age (Gao and Hollyfield, 1992). This later study suggested that the variation in the density of rod with age was not linear. Rod loss appeared to be more pronounced between the second and fourth decade than between the fourth and ninth decade. Gao and Hollyfield (1992) found that the ratio of photoreceptor to RPE cells did not vary significantly with age, indicating a parallel loss of these apposed cells. Loss of RPE cells in the far periphery is associated with variations in their typical morphology that are similar to those observed in the macular retina. In contrast, the morphology of RPE cells in the mid-periphery appears to remain stable throughout adulthood (Bhatia et al., 2016). Age-related changes in the structure of Bruch’s membrane are not as apparent in the peripheral retina as in the macular region. Changes in non-collagen protein content (Karwatowski et al., 1995) including lipids (Johnson et al., 2007) are not systematically observed. The reduction in hydraulic conductivity is less pronounced as compared to the macula (Moore et al., 1995), and macromolecular diffusion is only reduced by 66% between the first and ninth decade (Hussain et al., 2010).

The extension of dendritic fibers of a subtype of bipolar cells beyond the outer plexiform layer and into the outer nuclear layer has been observed in aged peripheral retinas (Eliasieh et al., 2007). Widespread loss of retinal capillaries with age has been observed in the peripheral retina (Kuwabara et al., 1961; Kuwabara and Cogan, 1965), as well as an increased incidence of vacuole-like structures in capillary basement membranes (Hogan and Feeney, 1963b; Powner et al., 2011); although the significance of this later finding is unclear. The ratio between the density of cells in the ganglion cell layer and that of rods does not vary significantly with age. Similarly to rods, loss of cells within the ganglion cell layer may be more pronounced between the second and fourth decade than between the fourth and ninth decade (Gao and Hollyfield, 1992). This study and others (Curcio et al., 1993) support the idea that ganglion cell losses in the peripheral retina occur at a rate smaller than that seen in the macula, although this has been contested (Harman et al., 2000). Curcio et al. (1993) identified a region of the peripheral retina displaying consistently lower ganglion cell densities in older eyes as compared to younger ones.

### Systemic Factors

It is beyond the scope of this review to describe systemic changes associated with aging; however, their effect on retinal mass transport systems should not be overlooked. It has long been known that cardiac output decreases with age while blood pressure increases (Boss and Seegmiller, 1981). Plasma proteome also displays marked variations with age (Lehallier et al., 2019). Serum or plasma proteins influence transport across endothelial cells (by modulating oncotic pressures for instance) (Bhave and Neilson, 2011) and semi-permeable membranes, and are therefore a key determinant of retinal homeostasis. The effect of these changes on retinal and choroidal blood flow is yet to be fully explored. Basic theoretical considerations suggest that a reduction in cardiac output and/or an increase in arterial blood pressure may exacerbate spatial heterogeneities in mass transfers between the choriocapillaris and outer retina (Zouache et al., 2016c). This effect is more pronounced for larger molecules, which have a comparatively longer travel time between blood and tissue. Whether the retinal and choroidal vasculatures have evolved mechanisms to adapt to these changes is unknown.

### Conclusion

The effect of aging on the retina is spatially heterogeneous; it is more prominent in the region with the highest energy requirements, the macula, and in the outer retina and choroid as compared to the inner retina. Direct and indirect evidence point to a progressive breakdown of mass transfers between retinal neurons and their respective metabolite delivery systems. However, the contribution of this breakdown to retinal aging and its correlation with cellular alterations and potential loss of function remains to be fully elucidated. The 30% loss in rod density in the macula appears to concord with the 34% decrease in choriocapillaris density observed with age (see Figure 5B). However, more work is necessary to understand the relation between these two systems. The largest change in retinal mass transport systems is by far seen in Bruch’s membrane. The selectivity of this barrier to transport is dramatically altered in aged eyes, with the transfer of larger molecules and fluids being comparatively more affected. Changes seen in the inner retina appear to be largely independent from those observed among photoreceptors and choroid; however, more work is necessary to confirm this.

## Conclusion and Perspectives

Our ability to fully assess correlations between variations in retinal neuron populations and changes in their respective mass transport systems using published data is limited by the fact that the key components to consider – neurons, vasculatures, extravascular and extracellular elements – have predominantly been studied in isolation. The retina involves many cellular elements that function through *symbiotic* relationships. It is now thought that photoreceptors are at the center of a metabolic landscape involving RPE and Müller cells (Kanow et al., 2017; Viegas and Neuhauss, 2021). These functional units rely on adequate mass exchange between Müller cells and retinal capillaries, and between choriocapillaris, Bruch’s membrane and RPE. Investigating these entities as units may yield better insights not only into interaction between retinal cells and mass transport systems, but also into the chain of events driving retinal senescence and vitreoretinal disorders.

Our assessment of the relation between retinal neuron populations and mass transport systems in aging is considerably limited by the lack of models or frameworks developed to differentiate between structural and functional changes caused by aging and those driven by disease. This limitation is more broadly a significant impediment to the study of retinal aging. A large part of our understanding in this regard comes from samples selected based on the absence of phenotypes traditionally associated with vitreoretinal disorders. But many (Zouache et al., 2020; Williams et al., 2021) have shown that defining retinal diseases using phenotype alone is not sufficient. The consequences of genetic mutations driving AMD is for instance often detected late in life; however, their effect on retinal and choroidal structures is likely to begin much earlier. An effective way to address this is to stratify subjects base on genetic susceptibility for disease (Pappas et al., 2021) and to exclude those at high risk from cross-sectional and longitudinal analyses.

Future studies may benefit from considering variations in mass exchange within the retina and their effect at the molecular, cellular and tissue levels. Models developed within frameworks centered around basic physical principles can provide extremely valuable insights into processes driving alterations and help generate hypotheses to be experimentally tested. A benefit of these approaches is the ability to link phenomenon occurring at distinct scales. Biological systems such as the retina are intrinsically multiscale, and cells themselves must integrate several scales to functions (Lecca, 2013). Integrating information from different levels to build a better understanding of the retina is a major challenge; however, it is key to designing effective strategies to slow or even reverse vision losses caused by aging and vitreoretinal disorders.

Laying down a framework capable of assessing the effect of variations in retinal neuron populations and their respective mass transport systems on visual function is incredibly challenging, in part because of the broad range of scales associated with the processes and structure at play. Such a framework is however necessary to determine the nature of the relation between cellular and tissue-level changes and vision. Alterations in the selective permeability of Bruch’s membrane restrict the movement of many molecules essential to the visual cycle; however, their effect on visual function is presently impossible to assess directly. Some have proposed that rod visual function is unlikely to be affected by rod losses observed in aging (Oyster, 1999). This would indicate that the relation between photoreceptor loss and visual impairment is not linear. Integrating functional and structural information from multiple scales will be necessary in future studies to understand the full extent of the resilience and adaptability of our visual system.

## Author Contributions

MAZ performed all literature searches, wrote and revised the review and generated all figures.

## Conflict of Interest

The author declares that the research was conducted in the absence of any commercial or financial relationships that could be construed as a potential conflict of interest.

## Publisher’s Note

All claims expressed in this article are solely those of the authors and do not necessarily represent those of their affiliated organizations, or those of the publisher, the editors and the reviewers. Any product that may be evaluated in this article, or claim that may be made by its manufacturer, is not guaranteed or endorsed by the publisher.
