# Supplementary material for: Variability in Retinal Neuron Populations and Associated Variations in Mass Transport Systems of the Retina in Health and Aging
Source: Front Aging Neurosci. 2022 Feb 25;14:778404. doi: 10.3389/fnagi.2022.778404 (PMC8914054; doi:10.3389/fnagi.2022.778404)
Supplement: Supplementary file 1 [file Table_1.docx]

| **Element** | **Figure 4**  **Panel** | **Property** | **Region** | **Reference** |
| --- | --- | --- | --- | --- |
| Rod Photoreceptors | B | Cell Density | Fovea to Periphery | (Curcio et al., 1990) |
| Cone Photoreceptors | B | Cell Density | Fovea to Periphery | (Curcio et al., 1990) |
| Ganglion Cells | D | Cell Density | Fovea to Periphery | (Curcio and Allen, 1990) |
| Bipolar Cells | F | Cell Density  (based on ratio relative to rods and cones) | Fovea to Periphery | (Martin and Grünert, 1992)  (Grünert et al., 1994) |
| Retinal Capillaries | H | Vascular Density | Perifovea | (Snodderly et al., 1992) |
|  |  |  | Peripapillary Region | (Snodderly et al. 1992 |
| Retinal Pigment Epithelium | C | Cell Density | Optic Nerve to Temporal Periphery | (Bhatia et al., 2016) |
|  |  |  | Optic Nerve to Nasal Periphery | (Panda-Jonas et al., 1996) |
| Choriocapillaris | E | Vascular Density | Subfoveal Region | (Ramrattan et al., 1994) |
|  |  |  | Periphery | (Spraul et al., 1999)  (Spraul et al., 2002) |
| Choriocapillaris | E | Vascular Pattern | Subfoveal Region | (Yoneya and Tso, 1987) |
|  |  |  | Equatorial Region | (Yoneya and Tso, 1987) |
|  |  |  | Periphery | (Yoneya and Tso, 1987) |
| Bruch’s Membrane | G | Thickness | Subfoveal Region | (Ramrattan et al., 1994)  (Nakaizumi, 1964) |
|  |  |  | Peripapillary Region | (Salzman, 1912)  (Garron, 1963)  (Nakaizumi, 1964) |
|  |  |  | Equatorial Region | (Nakaizumi, 1964) |
|  |  |  | Periphery | (Nakaizumi, 1964) |
| Bruch’s Membrane | I | Hydraulic Conductivity | Subfoveal Region | (Moore et al., 1995) |
|  |  |  | Periphery | (Moore et al., 1995) |

**References**

Bhatia, S. K., Rashid, A., Chrenek, M. A., Zhang, Q., Bruce, B. B., Klein, M., Boatright, J. H., Jiang, Y., Grossniklaus, H. E., and Nickerson, J. M. (2016). Analysis of RPE morphometry in human eyes. *Mol. Vis.* 22, 898–916.

Curcio, C. A., and Allen, K. A. (1990). Topography of ganglion cells in human retina. *J. Comp. Neurol.* 300, 5–25. doi:10.1002/cne.903000103.

Curcio, C. A., Sloan, K. R., Kalina, R. E., and Hendrickson, A. E. (1990). Human photoreceptor topography. *J. Comp. Neurol.* 292, 497–523. doi:10.1002/cne.902920402.

Garron, L. K. (1963). The Ultrastructure of the Retinal Pigment Epithelium with Observations on the Choriocapillaris and Bruch’s Membrane. *Trans Am Ophthalmol Soc* 61, 545–588.

Grünert, U., Martin, P. R., and Wässle, H. (1994). Immunocytochemical analysis of bipolar cells in the macaque monkey retina. *J. Comp. Neurol.* 348, 607–627. doi:10.1002/cne.903480410.

Martin, P. R., and Grünert, U. (1992). Spatial density and immunoreactivity of bipolar cells in the macaque monkey retina. *J. Comp. Neurol.* 323, 269–287. doi:10.1002/cne.903230210.

Moore, D. J., Hussain, A. A., and Marshall, J. (1995). Age-related variation in the hydraulic conductivity of Bruch’s membrane. *Invest. Ophthalmol. Vis. Sci.* 36, 1290–1297.

Nakaizumi, Y. (1964). The ultrastructure of bruch’s membrane. I. Human, Monkey, Rabbit, Guinea Pig, and Rat eyes. *Arch. Ophthalmol.* 72, 380–387. doi:10.1001/archopht.1964.00970020380016.

Panda-Jonas, S., Jonas, J. B., and Jakobczyk-Zmija, M. (1996). Retinal pigment epithelial cell count, distribution, and correlations in normal human eyes. *Am. J. Ophthalmol.* 121, 181–189. doi:10.1016/s0002-9394(14)70583-5.

Ramrattan, R. S., van der Schaft, T. L., Mooy, C. M., de Bruijn, W. C., Mulder, P. G., and de Jong, P. T. (1994). Morphometric analysis of Bruch’s membrane, the choriocapillaris, and the choroid in aging. *Invest. Ophthalmol. Vis. Sci.* 35, 2857–2864.

Salzman, M. (1912). *The Anatomy and Histology of the Human Eyeball in the Normal State, Its Development and Senescence*. Trans by E. V. L. Brown, The University of Chicago Press.

Snodderly, D. M., Weinhaus, R. S., and Choi, J. C. (1992). Neural-vascular relationships in central retina of macaque monkeys (Macaca fascicularis). *J. Neurosci.* 12, 1169–1193.

Spraul, C. W., Lang, G. E., Grossniklaus, H. E., and Lang, G. K. (1999). Histologic and morphometric analysis of the choroid, Bruch’s membrane, and retinal pigment epithelium in postmortem eyes with age-related macular degeneration and histologic examination of surgically excised choroidal neovascular membranes. *Surv Ophthalmol* 44 Suppl 1, S10-32.

Spraul, C. W., Lang, G. E., Lang, G. K., and Grossniklaus, H. E. (2002). Morphometric changes of the choriocapillaris and the choroidal vasculature in eyes with advanced glaucomatous changes. *Vision Res.* 42, 923–932. doi:10.1016/s0042-6989(02)00022-6.

Yoneya, S., and Tso, M. O. (1987). Angioarchitecture of the human choroid. *Arch. Ophthalmol.* 105, 681–687. doi:10.1001/archopht.1987.01060050099046.
