# Supplementary material for: Variability in Retinal Neuron Populations and Associated Variations in Mass Transport Systems of the Retina in Health and Aging
Source: Front Aging Neurosci. 2022 Feb 25;14:778404. doi: 10.3389/fnagi.2022.778404 (PMC8914054; doi:10.3389/fnagi.2022.778404)
Supplement: Supplementary file 2 [file Table_2.docx]

| **Element** | **Property** | **Region** | **Age Range** | **Variation** | **Reference** |
| --- | --- | --- | --- | --- | --- |
| Rod Photoreceptors | Cell Density  (per mm^2^) | Parafovea | 27-90 | $722,730-3417\times\mathrm{Age}$ | (Curcio, 2001) |
|  |  | Equator | 27-90 | $98,510-255\times\mathrm{Age}$ | (Curcio et al., 1993) |
|  |  | Equator | 18-95 | $138,110-571\times\mathrm{Age}$ | (Gao and Hollyfield, 1992) |
| Cone Photoreceptors | Cell Density  (per mm^2^) | Fovea | 27-90 | $217,900-716\times\mathrm{Age}$ | (Curcio et al., 1993) |
|  |  | Fovea | 18-95 | $190,290-326\times\mathrm{Age}$ | (Gao and Hollyfield, 1992) |
|  |  | Equator | 18-95 | $5,218-16\times\mathrm{Age}$ |  |
| Retinal Pigment Epithelium | Cell Density  (per mm^2^) | Fovea | 18-95 | $7,877-9\times\mathrm{Age}$ | (Gao and Hollyfield, 1992) |
|  |  | Equator | 18-95 | $5531-14\times\mathrm{Age}$ |  |
| Ganglion Cell Layer | Cell Density  (per mm^2^) | Equator | 18-95 | $1,097-5.65\times\mathrm{Age}$ | (Gao and Hollyfield, 1992) |
| Bruch’s Membrane | Thickness  (µm) | Macula | 6-100 | $2+0.027\times\mathrm{Age}$ | (Ramrattan et al., 1994) |
|  | Macromolecular Permeability  (µg/mm^2^/24 hours) | Macula | 9-85 | $270.545-3.027\times\mathrm{Age}$ | (Moore and Clover, 2001) |
|  | Hydraulic Permeability  (m/sec/Pa) | Macula | 9-85 | $2.2+317.1\times exp(-0.0727\times\mathrm{Age})$ | (Moore et al., 1995) |
|  |  | Periphery | 9-85 | $272.2\times exp(-0.046\times\mathrm{Age})$ |  |
| Choriocapillaris | Vascular Density | Fovea | 6-100 | $0.75-0.0034\times\mathrm{Age}$ | (Ramrattan et al., 1994) |
|  | Cross-sectional Lumen Diameter (µm) | Fovea | 6-100 | $9.85-0.033\times\mathrm{Age}$ |  |

**References**

Curcio, C. A. (2001). Photoreceptor topography in ageing and age-related maculopathy. *Eye (Lond.)* 15, 376–383. doi:10.1038/eye.2001.140.

Curcio, C. A., Millican, C. L., Allen, K. A., and Kalina, R. E. (1993). Aging of the human photoreceptor mosaic: evidence for selective vulnerability of rods in central retina. *Invest. Ophthalmol. Vis. Sci.* 34, 3278–3296.

Gao, H., and Hollyfield, J. G. (1992). Aging of the human retina. Differential loss of neurons and retinal pigment epithelial cells. *Invest. Ophthalmol. Vis. Sci.* 33, 1–17.

Moore, D. J., and Clover, G. M. (2001). The effect of age on the macromolecular permeability of human Bruch’s membrane. *Invest. Ophthalmol. Vis. Sci.* 42, 2970–2975.

Moore, D. J., Hussain, A. A., and Marshall, J. (1995). Age-related variation in the hydraulic conductivity of Bruch’s membrane. *Invest. Ophthalmol. Vis. Sci.* 36, 1290–1297.

Ramrattan, R. S., van der Schaft, T. L., Mooy, C. M., de Bruijn, W. C., Mulder, P. G., and de Jong, P. T. (1994). Morphometric analysis of Bruch’s membrane, the choriocapillaris, and the choroid in aging. *Invest. Ophthalmol. Vis. Sci.* 35, 2857–2864.
